# Protein carbamylation in atherosclerotic plaques correlates with uremia and disease progression, localizing predominantly to foam cells

**DOI:** 10.3389/fimmu.2025.1532250

**Published:** 2025-05-28

**Authors:** Valeria Saar-Kovrov, Aleksandra Pawlowska, Adrien Guillot, Marion J. J. Gijbels, Judith C. Sluimer, Lieve Temmerman, Pieter Goossens, Barend M. E. Mees, Frank Tacke, Vera Jankowski, Joachim Jankowski, Marjo M. P. C. Donners, Erik A. L. Biessen

**Affiliations:** ^1^ Department of Pathology, Cardiovascular Research Institute Maastricht, Maastricht University Medical Center, Maastricht, Netherlands; ^2^ Department of Hepatology and Gastroenterology, Charité - Universitätsmedizin Berlin, Campus Virchow-Klinikum and Campus Charité Mitte, Berlin, Germany; ^3^ School for Oncology and Developmental Biology (GROW), Maastricht University Medical Center, Maastricht, Netherlands; ^4^ Department of Medical Biochemistry, Experimental Vascular Biology, Amsterdam University Medical Centers, University of Amsterdam, Amsterdam, Netherlands; ^5^ Centre for Cardiovascular Science, University of Edinburgh, Edinburgh, United Kingdom; ^6^ Department of Surgery, Maastricht University Medical Center (UMC)+, Maastricht, Netherlands; ^7^ Institute for Molecular Cardiovascular Research (IMCAR), Rheinisch-Westfälische Technische Hochschule (RWTH) Aachen University, Aachen, Germany

**Keywords:** atherosclerosis, macrophages, foam cells, carbamylation, kidney disease

## Abstract

**Introduction:**

Carbamylation is a non-enzymatic post-translational protein modification common in patients with uremia that causes pro-atherogenic alterations in plasma proteins. It is abundantly present in late-stage atherosclerotic plaques; however, the pathogenic relevance and functional consequences of this accumulation are not known.

**Methods:**

Human atherosclerotic plaque tissue samples were stratified by plaques’ stage and kidney function.

**Results:**

Immunohistochemistry revealed a significantly higher carbamylated lysine (carb-lys) abundance in latestage hemorrhaged plaques of chronic kidney disease patients compared to early-stage plaques, and a significant negative correlation to glomerular filtration rate for the advanced plaques. While we saw the difference in the total levels of carbamylation between early and advanced plaques, cellular carbamylation signal, studied in a parallel cohort of stable vs unstable plaques, did not differ between plaque stages but significantly correlated to CD68, PLIN2, and LGALS3 signals. Functional effects of carbamylated LDL (carbLDL) uptake on macrophages were studied *in vitro* on an in-house developed confocal-based microscale multi-assay platform to screen multiple cellular functions and demonstrated similar foam cell formation compared to the uptake of oxidized LDL (oxLDL). However, in contrast to oxLDL, carbLDL did not induce PPARg reporter gene expression, suggesting differential capacity to induce lipogenic pathways. Moreover, unlike oxLDL, carbLDL did not induce apoptosis or ROS production.

**Discussion:**

Taken together, our findings demonstrate an accumulation of carbamylated protein during plaque progression in patients with reduced kidney function. This can be, at least partially, explained by uptake of carbLDL particles by the macrophages. CarbLDL uptake, in turn, can induce foam cell formation but seems less cytotoxic than oxLDL.

## 1 Introduction

Excessive formation of harmful post-translational modifications (PTMs) of proteins is a common feature of systemic disorders such as rheumatoid arthritis, diabetes mellitus, chronic kidney disease (CKD) and others. A well-known example is oxidation of low-density lipoprotein (LDL), which is shown to induce inflammation, lipid accumulation and cell death in atherosclerosis (1).

Another example of an undesirable PTM, found in increased levels in patients with CKD, yet much less studied, is carbamylation (2). Carbamylation originates from non-catalyzed reaction between free amino groups of protein amino acids, e.g. lysine, arginine and the N-terminal amine, and isocyanate anion [OCN]^-^ originating from spontaneous deamination of urea (3) or oxidation of thiocyanate by myeloperoxidase (MPO) (4, 5). Lysine carbamylation gives rise to carbamyl-lysine, or homocitrulline, which is suggested as a biomarker for total carbamylation levels in chronic renal failure (6). Moreover, carbamylation is known to cause potentially pro-atherogenic alterations in plasma proteins and is significantly associated with increased mortality in patients with CKD (2, 7, 8). For instance, carbamylation was shown to increase stiffness of the elastic fibers (9), disrupt the interaction between cells and plasma proteins (9–11), as well as alter fibrin polymerization kinetics affecting its structure and stability (11). Additionally, carbamylated high-density lipoprotein (HDL) was shown to lose its anti-inflammatory and reverse-cholesterol transport properties (4, 12, 13), while carbamylated LDL is known to induce foam cell formation (5, 14, 15), smooth muscle cells adhesion and proliferation (16, 17), as well as endothelial dysfunction (8, 14, 18, 19). However, the evidence on direct effects of protein carbamylation on, in particular, macrophage mediated inflammation and atherosclerosis is scarce.

While atherosclerotic plaques in patients with normal kidney function showed a dramatic increase in total levels of carbamylation (12, 20), to the best of our knowledge, there is no data on plaque carbamylation levels in patients with kidney insufficiency reported in the literature thus far.

Therefore, in this study we investigated protein carbamylation in atherosclerotic plaque progression in uremic patients. Additionally, we mapped the cellular carbamylation levels with known foam cell markers and investigated the functional implications of LDL carbamylation on human THP-1 derived macrophages *in vitro* using oxLDL as a benchmark.

## 2 Materials and methods

### 2.1 Atherosclerotic plaque samples

Atherosclerotic plaque samples were obtained during carotid endarterectomy as described earlier (21). Plaque samples were cut into parallel, transverse 5-mm thick segments. Alternating segments were fixed for 24 hours in formalin then decalcified for 4 hours, processed, and embedded in paraffin. Plaque classification was performed on hematoxylin-eosin (H&E) stained 4-µm thick slides (22) and the first, plaque progression cohort was assembled based on the plaque types: 1) pathological intima thickening (PIT; early), 2) fibrous cap atheroma (FCA; stable plaque) and 3) lesions with intraplaque hemorrhage (IPH; advanced culprit lesion), with n= 8–9 per group. Study groups were matched for patients’ age and sex (**Supplementary Table S1**).

In an independent cohort, plaque samples were stratified based on the patients’ estimated glomerular filtration rate (eGFR) (23) applying a threshold value of 60 mL/min/1.73 m^2^: patients with eGFR< 60 being assigned to the CKD group, and patients with eGFR> 60 values to the non-CKD group. Plaque samples were also classified into PIT, FCA, and IPH within the groups (**Supplementary Table S2**), resulting in 10 CKD (10 PIT, 5 FCA, and 10 IPH) and 9 non-CKD samples (9 PIT, 4 FCA, and 7 IPH).

As a third cohort, the MaasHPS plaque cohort was used, of which details are described elsewhere (21). Within MaasHPS we defined a two-armed subcohort: one group including non-hemorrhaged “stable” plaques (intima thickening, thick and thin FCA; n= 10) and a second group including hemorrhaged “unstable” plaques (IPH; n= 8), with no significant differences in patients’ sex or age between the groups (**Supplementary Table S3**).

This work conforms to the ethical norms and standards in the Declaration of Helsinki. All participants have given informed written consent prior to the inclusion. All patient material was collected in accordance with the Dutch Code for Proper Secondary Use of Human Tissue (https://www.federa.org) and the local Medical Ethical Committee (protocol number 16-4-181).

### 2.2 Carbamylation immunohistochemistry

Cross-sections (4-μm) of paraffin embedded plaques were treated with 0.3% H_2_O_2_ to block endogenous peroxidase and subjected to heat induced antigen retrieval using a low pH target retrieval solution (DAKO, Agilent Technologies). Carbamylation staining was performed with rabbit polyclonal anti-carbamyl lysine antibody (1:8000; STA-078, CellBiolabs) using Brightvision poly-HRP anti-rabbit IgG (ImmunoLogic) as secondary antibody, developed with chromogen 3,3′-diaminobenzide (DAB; Dako) and counterstained with hematoxylin. Scans were analyzed with QuPath v0.4.0., plaque area was selected including all intima from the internal elastic lamina toward the lumen and percentage of DAB positive area was analyzed using thresholding.

Anti-carbamyl lysine antibody specificity was validated as described previously (20) using 1 hour pre-incubated with 10 µg/ml of carbamylated bovine serum albumin (BSA). Antibody diluted in non-modified BSA solution served as a control. After that, the staining process was performed as described above and slides were imaged on a bright field microscope using x10 and x40 objectives (**Supplementary Figures S1A–D**).

MPO-carbamylation double staining was performed on advanced plaque samples (FCA) using monoclonal mouse anti-human MPO antibody (1:60; MAB3174; R&D Systems) and anti-carbamyl lysine antibody, as described above. Then, sections were incubated with Brightvision poly-HRP anti-rabbit and Brightvision poly-AP anti-mouse IgG mix (1:1) and the signals were visualized with Vector Red (Vector Laboratories) and DAB, according to the manufacturers’ protocol. Finally, the slides were counterstained with hematoxylin and visualized on an upright fluorescence microscope (Leica DM4000) equipped with Nuance FX camera (Perkin Elmer) as described previously (24) using x40 objective. Co-localization of the signals was analyzed in Fiji (25) using JACoP plugin (26).

### 2.3 Sequential immunofluorescent analysis

#### 2.3.1 Staining

Sequential immunofluorescent (IF) staining was performed as described before (27). Briefly, paraffin embedded plaque tissue slides were deparaffinized and antigen retrieval was performed, as described above. Auto-fluorescent signal was removed by incubating the slides in phosphate-buffered saline (PBS) containing 4.5% (w/v) H_2_O_2_ and 20mM NaOH for 90 minutes at room temperature, as described previously (28). Slides were then blocked with 2% normal goat serum (Thermo Fisher Scientific) and incubated with the anti-carbamyl lysine antibody (1:1000; STA-078; CellBiolabs) and rat anti-LGALS3 (1:1000; 125402, BioLegend). Goat-anti-rabbit (1:500; Alexa Fluor 750; A21039, Thermo Fisher Scientific) and goat-anti-rat (1:500; Alexa Fluor 647; 4418S; Cell Signaling) were used as secondary antibodies. Nuclei were stained with 4′,6-diamidino-2-phenylindole (DAPI) (Sigma-Aldrich). Slides were mounted with VectaMount AQ Aqueous Mounting Medium (Vector BioLabs) and immediately scanned on Zeiss Axio Observer 7 using the 20x objective. Before the second round of staining, the mounting was removed with distilled water and slides were stripped in 62.5 mM Tris-HCl pH 6.8 (Bio-Rad), 2% (w/v) SDS (Rockland Immunochemicals) and 114.4 mM β-mercaptoethanol (Sigma-Aldrich) in distilled water at 56°C for one hour and then extensively washed in PBS containing 1% Tween-20. Then, the staining was repeated as above using mouse-anti-human CD68 antibody (1:100; M0814; Agilent) and rabbit-anti-human PLIN2 (1:50; NB110-40877; Novus Biologicals). The secondary antibodies used were goat-anti-mouse (1:500; Alexa Fluor 647; 4410S; Cell Signaling) and goat-anti-rabbit (1:500; Alexa Fluor 750; A21039, Thermo Fisher Scientific). A control slide was used to validate the specificity of the secondary antibodies. Additionally, since both anti-carb-lys and anti-PLIN2 antibodies originate from the same host species, two adjacent plaque tissue samples were stained in parallel to confirm the staining patterns and eliminate the option of signal leaking (**Supplementary Figures S4N, O**).

#### 2.3.2 Image processing

Images were processed as described earlier (27, 29). Briefly, scanned images were first stitched, and background was removed using the default settings on the ZEISS software ZEN 3.1 (blue edition). The single-channel images acquired during the same cycle were combined into a hyperstack, keeping DAPI as channel 1. Images were checked between the cycles and damaged tissue areas were removed to improve the alignment outcome. All the hyperstacks were then concatenated, and alignment was performed using the FIJI HyperStackReg V5.6 plugin on the Affine setting and DAPI (channel 1) as a reference. After the alignment, channels were saved as individual images.

#### 2.3.3 Signal values extraction and cell classification

Cells were segmented in QuPath v.0.4.0. using Cell Detection command guided by the nuclear DAPI signals (cell expansion: 10 pixels). Only cells within the intima region were segmented. Then, the region of interest (ROI) was exported to Fiji and an image overlay was built for all of the channels. Signal information per cell, such as cell area, integrated density, skewness, as well as mean intensity for each channel was extracted into Excel sheet. Correlated total cell fluorescence (CTCF) of the channels was calculated for each cell as described before (30) using the following formula: *Integrated density – (Area × Mean intensity of the background).* For classification of the cells into positive/negative, a threshold for each signal was established based on the signal’s properties, such as skewness and CTCF values, by manual analysis of 100 cells of one sample and validation on a second sample. The threshold was kept the same throughout the analysis of all samples. Used thresholds are listed in **Supplementary Table S4**. PLIN2 signal was further stratified into three groups (cells with high signal, low, and negative) for higher phenotypic resolution: the high signal group showing values above the high threshold (average of all cell signal intensities plus one standard deviation value); low signal group – cells showing values between the standard and the high thresholds, and negative group with cells showing values below the standard threshold.

### 2.4 LDL isolation and modification

LDL was isolated from human plasma as described before using density gradient ultracentrifugation for 16 hours at 4°C (31). LDL sample protein concentration was determined with bicinchoninic acid kit (BCA; Pierce) and subsequently adjusted to 0.5 mg/ml with PBS. For oxidation, LDL was incubated with 0.32 mM CuSO_4_ overnight at 37°C after what the reaction was terminated by addition of 50 μM EDTA. For carbamylation, LDL was incubated with 0.1 M KOCN overnight at 37°C. Then, both reactions were dialyzed against PBS containing 10 μM EDTA for 24 hours. Modifications were confirmed using 2% agarose gel electrophoresis stained with Coomassie blue (**Supplementary Figure S1E**). Final LDL concentrations were measured again after the dialysis using BCA kit.

### 2.5 Cell assays

Human monocytic cells THP-1 (American Type Culture Collection (ATCC)) were cultured in RPMI 1640 medium (72400047, Gibco) with 10% heat-inactivated fetal calf serum (FCS; FBS-12A, Capricorn Scientific) and 1% Penicillin Streptomycin (P/S, 15070-063, Gibco).

Cells were seeded on either 96-well black optical imaging plates (BD<ns/>353219) for the functional assays (n= 8 per condition) at 40–000 cells/well or on 6-well cell culture plates at 1.2 *10^6^ cells/well (Greiner Bio-One) for carb-lys ELISA and RNA isolation (n= 4 per condition) and differentiated into macrophages with 200 nM phorbol 12-myristate 13-acetate (PMA; Sigma) for 72 hours. Functional assays were performed using a screening platform MacroScreen described previously (32, 33). The response was measured on the BD Pathway 855 (BD Bioscience) taking nine images per well that were stitched and subsequently analyzed using CellProfiler (34). Cells were then classified as positive/negative based on the mean cell fluorescence and results expressed in percentage of the total cell number, unless otherwise stated.

#### 2.5.1 Cell carbamylation treatment

For cell carbamylation analysis, fully differentiated human macrophages (THP-1-derived) were incubated with either 5 mM KOCN (Sigma Aldrich) or 40 mM Urea (Sigma) in complete RPMI medium for 24 hours.

For carbamylated protein uptake experiment, the cells were incubated with carbamylated and non-modified BSA (20 μg/ml, fatty acids-free; Sigma) for 24 hours. To induce carbamylation, 20 mg/ml BSA was incubated with 0.1 M KOCN overnight at 37°C and subsequently extensively dialyzed against PBS. BSA carbamylation was confirmed on agarose gel electrophoresis and dot blot (**Supplementary Figures S1E, F**).

#### 2.5.2 Lipid uptake assay

Differentiated THP-1-derived macrophages were incubated for 2.5 hours in complete RPMI medium containing 8 μg/ml oxLDL, carbLDL or non-modified LDL and 2 μg/ml Topfluor (Avanti Polar Lipids), prepared just prior to addition. After the incubation, Hoechst 33342 (Sigma) was added to stain the nuclei, then cells were washed with PBS and imaged using a 10x objective.

#### 2.5.3 Foam cell formation

Foam cell formation was induced on differentiated THP-1 -derived macrophages using 24-hour incubation with either oxLDL, carbLDL or non-modified LDL at the concentration of 20 μg/ml each in FCS-free RPMI medium. After that, cells were fixed in 10% formalin for 10 min at room temperature, and primed with 60% isopropanol for 15 min. Then, cells were stained with 3 mg/ml Oil Red O (Merck) solution for 20 min, washed with 60% isopropanol, counterstained with hematoxylin and visualized with 40x objective. The number of droplets was quantified by counting the visible droplets divided by the number of cells in the field of view in three different areas to get the average number of droplets per cell. Four wells were analyzed per condition.

#### 2.5.4 Apoptosis

Foam cells were induced as specified above. Without any additional stimulation, cells were stained with Hoechst, washed with annexin binding buffer (10 mM 4-(2-hydroxyethyl)-1-piperazineethanesulfonic acid, 140 mM NaCl and 5 mM CaCl_2_; pH of 7.4) and incubated with 2.5 ng/ml Annexin-V-OG (FP488) (35) for 15 minutes. The plate was then washed with annexin binding buffer and imaged using a 10x objective.

#### 2.5.5 ROS production

Foam cells, generated as described above, were treated with 20 µM 2′,7′-dichlorodihydrofluorescein diacetate (DCFDA; Sigma) in Opti-MEM (Reduced Serum Medium, Gibco) containing 0.5% FCS for 1 hour at 37°C. After that, cells were treated with 50 ng/ml PMA for one additional hour. Cells were then incubated with Hoechst 33342 (Sigma), washed with KI quencher solution (2 mM KH_2_PO_4_, 0.2 M KI) and visualized using a 10x objective.

#### 2.5.6 Phagocytosis

Cells of the technical control were incubated with 25 μM cytochalasin D (Sigma) for 30 minutes at 37°C. Then, medium was removed, and all cells were incubated with 12.5 μg/ml of pHrodo-labeled Zymosan (Thermo Fisher Scientific) for 1 hour at 37°C. After that, the nuclei were stained with Hoechst, washed with PBS and imaged using a 10x objective.

### 2.6 Confocal imaging

Human primary macrophages were generated from peripheral blood mononuclear cells (PBMCs) as described previously (32). Briefly, PBMCs were isolated by Ficoll-Paque gradient centrifugation (Sigma) of leukocyte reduction system cones (Stem Cell Technologies) obtained from healthy blood donors at University Hospital RWTH Aachen, Germany. CD14^+^ cells were then purified using CD14 specific magnetic beads and LS column (Miltenyi) according to the manufacturers protocol. A pool of three donors was used for the experiment. Cells were cultured on Nunc™ Lab-Tek™ chambered coverglasses (Thermo Fisher Sientific). After differentiation using macrophage colony-stimulating factor (100 ng/ml) for 7 days, macrophages were treated with 50 μg/ml of carbamylated LDL for 24 hours. Cells were washed with PBS, fixed with 2% paraformaldehyde for 10 min, and permeabilized in PBS containing 0.1% Triton-X100. Cells were then washed with cell buffer (2 mM EDTA, 0.5% BSA w/v in PBS) and incubated first, with anti-carb-lys antibody (1:4000; STA-078, Cell Biolabs) for one hour, and subsequently with Alexa Fluor™ 488-coupled chicken anti-rabbit secondary antibody (1:1000; A-21441, Thermo Fisher Scientific) for 30 min with a washing step in between. After a washing step, cells were stained with DAPI to visualize nuclei and finally washed with PBS. Cells were then imaged on Leica TCS SP8 confocal microscope under x63 water immersion objective. The image of the cell was taken with an additional x4 zoom.

### 2.7 Dot blot

Dot blot was performed to confirm BSA modification as follows. Modified BSA was applied on nitrocellulose membrane at 1 µl per application containing 20, 2, 0.2, or 0.02 ng of protein and let to dry. The membrane was blocked with 1% BSA in PBS for 1 hour at room temperature under gentle agitation. After wash with 0.01% Tween 20 (Sigma Aldrich) in PBS, the membrane was incubated with primary ani-carb-lysine antibody (1:8000; STA-078; CellBiolabs) for one hour and subsequently with swine anti-rabbit Ig/HRP secondary antibody (1:1000; P0399; Dako) for one hour. Finally, the membrane was developed using SuperSignal West Femto Maximum Sensitivity Substrate (Thermo Fisher Scientific) and visualized on a digital scanner.

### 2.8 ELISA

After cell stimulation, media was removed, and cells were washed with PBS and lysed using RIPA buffer (50 mM Tris, 150 mM NaCl, 0.1% v/v NP-40, 0.5% w/v sodium deoxycholate, v/v 0.1% SDS; pH= 8) supplemented with protease inhibitors (Roche). Protein concentrations were determined with BCA assay (Pierce).

In-house developed carbamylation ELISA was performed as follows. Cell lysates were diluted 1:3 in PBS and incubated on 96-well Nunc MaxiSorp plate (Thermo Fisher Scientific) for 2 hours at room temperature. The plate was then washed with 0.05% Tween in PBS and blocked with 2% BSA in PBS for one hour. After washing, the plate was incubated with primary ani-carb-lysine antibody (1:3000; STA-078; CellBiolabs) for two hours and subsequently with swine anti-rabbit Ig/HRP secondary antibody (1:1000; P0399; Dako) for one hour at room temperature. Signal was visualized with KPL TMB Peroxidase Substrate (SeraCare). Sample values were normalized to their protein concentrations.

TNFα ELISA was performed on 1:5 diluted cell medium after lipopolysaccharide (LPS) stimulation (1 ng/ml for 6 hours) according to the manufacturer’s protocol (R&D Systems).

### 2.9 Mass-spectrometry

After cell stimulation, media was removed, and cells were washed with PBS and lysed using RIPA buffer (50 mM Tris, 150 mM NaCl, 0.1% v/v NP-40, 0.5% w/v sodium deoxycholate, v/v 0.1% SDS; pH= 8) without protease inhibitors. Protein concentrations were determined with BCA assay (Pierce) and equal protein amounts were separated on a 4-20% gradient SDS-PAGE. Gels were stained with Coomassie blue and destained with 10% acetic acid-25% methanol. The two most abundant protein bands (β-actin and α-tubulin) were cut from the gel (~38–42 kD and 50–55 kD, resp.). The gel plugs were manually separated, washed and equilibrated, by using ammonium bicarbonate in acetonitrile. The isolated proteins were digested and analyzed by matrix-assisted-laser-desorption/ionization-time of flight-mass spectrometer (MALDI-TOF-TOF) (36). Briefly the protein plugs were incubated with ammonium bicarbonate (50mmol^-1^) and 0.03% w/C trypsin for 24h at 37°C, desalted and concentrated by ZipTip_C18_ technology (Millipore, Billerica, MA, USA) and eluted with 80% acetonitrile directly onto the (MALDI) target plate (MTP-Ground steel 400/384; Bruker Daltonics) using alpha-cyano-4-hydrocinnamic acid as MALDI matrix and C13. The subsequent mass-spectrometric (MS) analyses were performed using both a MALDI-time of flight/time of flight (TOF/TOF) mass spectrometer (Ultraflex; Bruker-Daltonic, Germany) and a MALDI-Rapiflex mass-spectrometer (Bruker-Daltonics, Germany). The MALDI-TOF/TOF instruments were equipped with a smart-beam laser operated at a repetition rate of 100–200 Hz. The presented spectra (**Supplementary Figure S6**) are the representative average of 1000 single-shot spectra in MS mode. Mass-spectra were analyzed in reflector mode with delayed ion-extraction, while MS/MS fragments were analyzed using Lift-option of the MALDI-TOF/TOF mass spectrometer. Calibrated and annotated spectra were subsequently subjected to a Swiss-Prot database search (http://www.expasy.org/) utilizing the software tool “Bruker Bio-Tool 3.2 and the “Mascot 3.0 search engine” (Matrix Science Ltd, London, UK).

### 2.10 Quantitative PCR

RNA was isolated using the TRIzol reagent (Thermo Fisher Scientific) and subsequent phase separation with chloroform following manufacturer’s protocol. The purity of RNA was analyzed on NanoDrop (NanoDrop Technologies^®^). Quantitative polymerase chain reaction (qPCR) was performed with 10 ng cDNA using SYBR Green Supermix (Bio-Rad). Analysis was performed on CFX Manager Software version 3.1 (Bio-Rad). Ubiquitin C gene was used as the housekeeper. A20 forward primer sequence: 5’- TGAGACATTGGAGGAGCTTT -3’; and reverse primer: 5’- TGCTTGTCACTGTCGGTAGAA -3’. IκBα forward primer: 5’- GGCCAGCTGACACTAGAAAAC -3’; and reverse primer: 5’- GTTAGAGCGCCGAAGGAGT -3’. CCL2 forward primer: 5’- GCAATCAATGCCCCAGTCAC -3’; and reverse primer: 5’- CTTGAAGATCACAGCTTCTTTGGG -3’. CD36 forward primer: 5’- AGTCACTGCGACATGATTAATGGT -3’; and reverse primer: 5’- CTGCAATACCTGGCTTTTCTCA -3’. MPO forward primer: 5’- CCAGATCATCACTTACCGGGA -3’; and reverse primer: 5’- CACTGAGTCATTGTAGGAACGG -3’. ABCA1 forward primer: 5’- AGGTTGCTGCTGTGGAAGAA -3’; and reverse primer: 5’- GCAGCAGCTGACATGTTTGT -3’. ABCG1 forward primer: 5’- CCTGTCTGATGGCCGCTTTC -3’; and reverse primer: 5’- CCTCATCCACCGAGACACAC -3’. PLIN2 forward primer: 5’- TCAGCTCCATTCTACTGTTCACC -3’; and reverse primer: 5’- CCTGAATTTTCTGATTGGCACT -3’.

### 2.11 Statistical analysis

Statistical analysis was performed on GraphPad Prism 8. For *in vitro* assay data, two group comparisons were analyzed with Student’s t test; and for plaque histology data – by Mann-Whitney U-test or Spearman’s correlation whenever applicable. Multiple variable data (n> 2) was analyzed using one-way ANOVA. All data are expressed in mean ± SD.

## 3 Results

### 3.1 Carbamylated protein abundance increases with plaque progression in patients with kidney insufficiency

**Figure 1:**
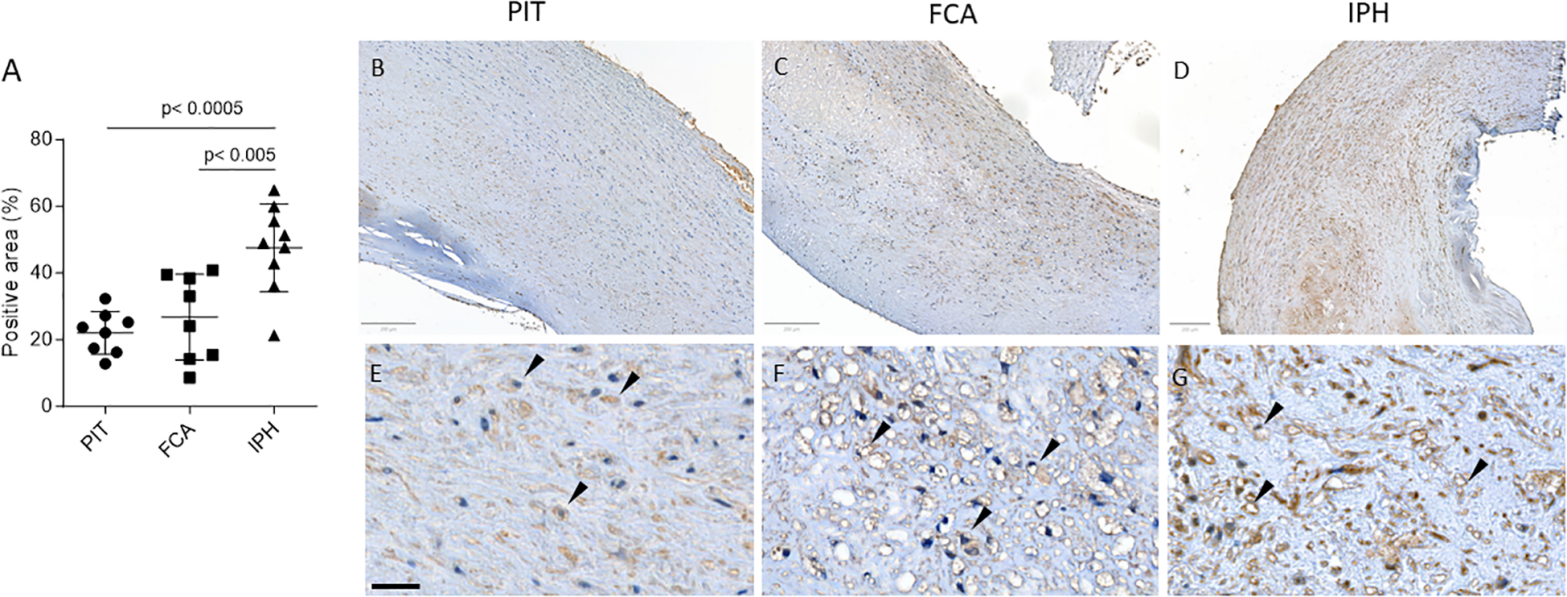
Total carb-lys positive area increases with plaque progression. **(A)** Quantification of total carb-lys positive area expressed in percentage of the total plaque area. PIT – pathological intima thickening (early plaque stage); FCA - fibrous cap atheroma (advanced stable plaque); IPH - intraplaque hemorrhage lesions (advanced ruptured plaque). **(B-G)** Representative images of PIT **(B, E)**, FCA **(C, F)**, and IPH **(D, G)** plaque samples stained for carb-lys. **(E-G)** Close up images demonstrating carb-lys positive foam cells (black arrows). Size bars – 200 (top panel) and 50 µm (bottom panel).

To assess the abundance of protein carbamylation in atherosclerotic lesions, immunohistochemistry (IHC) was performed on a carotid artery plaque cohort stratified for progression stage (i.e. pathological intimal thickening (PIT), Fibrous cap atheroma (FCA) and intraplaque hemorrhage (IPH); **Supplementary Table S1**) as described in the Methods section. We observed a significant stage-dependent increase in carb-lys positive area percentage (IPH vs PIT: 47.57 ± 13.13% vs 22.12 ± 6.41%, p< 0.0005; **Figures 1A–D**), which was present both intra- and extracellularly. Note that carb-lys content (already presented as percentage of total plaque area) significantly correlated to the total plaque area (**Supplementary Figures S2A, B**), suggesting that the increase in carbamylated protein in the plaque is associated with processes that mediate plaque progression, e.g. lipid accumulation, immune cell infiltration, necrosis or neovascularization. Indeed, IHC staining showed strong carb-lys signal in small foam cells, independently of the plaque stage (**Figures 1E–G**).

**Figure 2:**
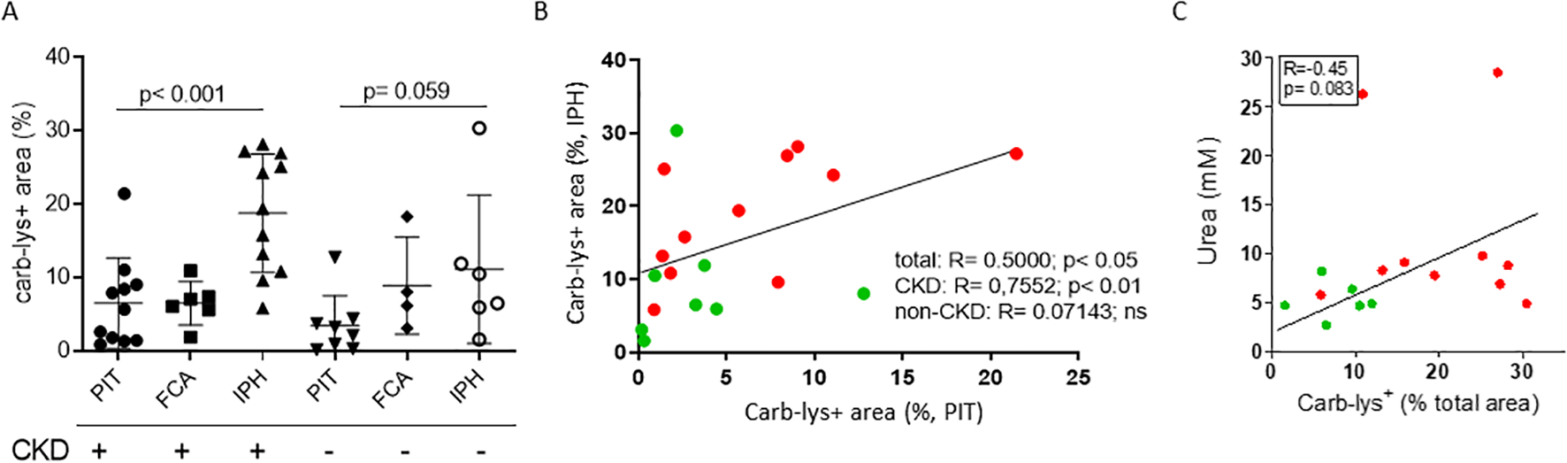
Plaque carb-lys positive area percentage correlates with patients’ kidney function decline. **(A)** Carb-lys+ area percentage of total plaque area in PIT, FCA and IPH plaques of patient with (eGFR< 60) and without CKD (eGFR> 60). **(B)** Spearman’s correlation plot showing significant positive correlation between carb carb-lys+ area percentage of PIT and IPH plaques coming from the same patient, and **(C)** moderate correlation between patients’ blood urea levels and carb-lys+ area percentage of their IPH plaque samples. CKD patient data points are in red and non-CKD – in green.

CKD patients are known to have increased propensity for carbamylation due to increased plasma urea (2). Indeed, in a second plaque cohort (CKD vs non-CKD, stratified based on patients’ eGFR values) only CKD patients showed increased plaque carb-lys staining of IPH compared to PIT plaques (IPH vs PIT: 19.69 ± 7.88% vs 6.38 ± 6.46%, p< 0.001; **Figure 2A**). We saw a trend toward higher carbamylation levels in IPH plaques of CKD patients compared to those of non-CKD patients, however the difference was not significant. As the cohort samples were paired (**Supplementary Table S2**), this allowed comparison of PIT to IPH plaque carbamylation content. Spearman’s rank test showed a significant positive correlation between patients’ PIT and IPH plaque carb-lys levels, however only for the total (ρ= 0.5000, p< 0.05) and CKD group (ρ= 0.7697, p= 0.01) but not for the non-CKD group (**Figure 2B**). Carb-lys signal abundance in IPH plaques showed a moderate correlation with patients’ blood urea levels (ρ= -0.45, p= 0.08; **Figure 2C**) but no correlation with any of the other patients’ parameters such as age, BMI, blood cholesterol and triglyceride levels, blood pressure, or arterial stenosis severity (**Supplementary Figure S3A**). Thus, plaque carb-lys levels appear to be patient-dependent and correlate to their kidney function. Of note, even though smoking was previously shown to increase levels of thiocyanate and induce protein carbamylation (5, 37, 38), there was no significant difference in plaque carb-lys levels between smokers and non-smokers in this cohort (**Supplementary Figures S3B, C**), although a higher-powered study would be required to draw any definite conclusions. Importantly, and in keeping with our initial findings (**Figures 1E–G**), for all plaque stages foam cells were seen to display particularly high carb-lys signal (data not shown).

### 3.2 Cellular carb-lys signal associates with foam cells independently of plaque type

**Figure 3:**
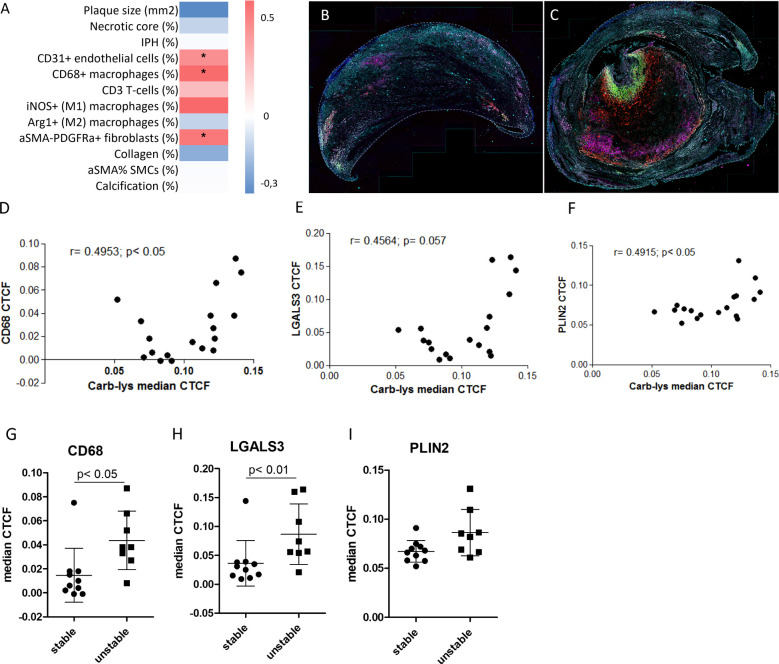
Carb-lys signal correlates with macrophage and foam cell markers. **(A)** Correlation-based heatmap showing correlation of carb-lys median CTCF values to the MaasHPS plaque traits with corresponding p values (**p< 0.01; *p< 0.05). **(B, C)** Representative merge images of a stable **(B)** and unstable **(C)** plaques stained for CD68 (red), LGALS3 (green), PLIN2 (magenta), carb-lys (cyan), and DAPI (blue). **(D-F)** Correlation plots showing carb-lys CTCF correlation to CD68 **(D)**, LGALS3 **(E)**, and PLIN2 **(F)** median CTCF. **(G, H)** Median CTCF values of CD68 **(G)**, LGALS3 **(H)**, and PLIN2 **(I)** comparing stable plaque group to unstable. Each point represents median cellular CTCF values per plaque sample (n= 18 total).

We next examined the association of plaque carbamylation levels with cellular and non-cellular plaque traits. Therefore, we stained a sub-cohort from the Maastricht Human Plaque Study (MaasHPS) (21) cohort, deeply phenotyped at morphology and IHC level, consisting of stable (FCA/PIT; n= 10) and unstable (IPH; n= 8) plaque samples (**Supplementary Table S3**) for carb-lys content. After image processing, extracted cellular carb-lys signal intensity values were normalized to obtain correlated total cell fluorescence (CTCF) and correlated to the plaque progression stage and traits. Interestingly, almost all detected cells showed some degree of carb-lys positivity while median CTCF values did not differ between stable and unstable plaques (**Supplementary Figure S4A**). In line with the earlier observed strong IHC carb-lys signal in foam cells (**Figures 1E–G**), we found that carb-lys staining intensity significantly correlated to the number of CD68^+^ cells (ρ = 0.594; p< 0.05; **Figure 3A**), with a stronger tendency to association with iNOS^+^ (M1-like) than with Arg1^+^ (M2-like) macrophages (ρ= 0.600 vs ρ= 0.055, respectively, ns). Interestingly, car-lys content showed a similar, significant correlation to CD31^+^ endothelial cells (ρ= 0.509, p< 0.05), confirming published data (39), and αSMA^-^PDGFR^+^ fibroblast (ρ= 0.565; p< 0.05), but not to αSMA+ smooth muscle cells (SMCs) content (ρ= 0.235, ns). Carb-lys median CTCF did not correlate to any of the other studied traits, including T-cell content, calcification, collagen content, or neovascularization.

To confirm the co-localization of carb-lys signal with foam cells, we performed sequential IF staining for macrophage (CD68) and foam cell markers, PLIN2 and LGALS3 (representative merged images are shown in **Figures 3B, C**; individual channel images in **Supplementary Figures S4F–M**). Indeed, carb-lys signal showed significant correlation with CD68 signal as well as with that of LGALS3 and PLIN2 (**Figures 3D–F**). Note that both CD68 and LGALS3 median CTCF and percentage positive cells were significantly increased in unstable compared to stable plaques, while PLIN2 showed a similar trend (**Figures 3G–I**; **Supplementary Figures S4B–E**), suggesting that the increased total carb-lys signal in IPH plaque could be attributable to the increased foam cell content.

**Figure 4:**
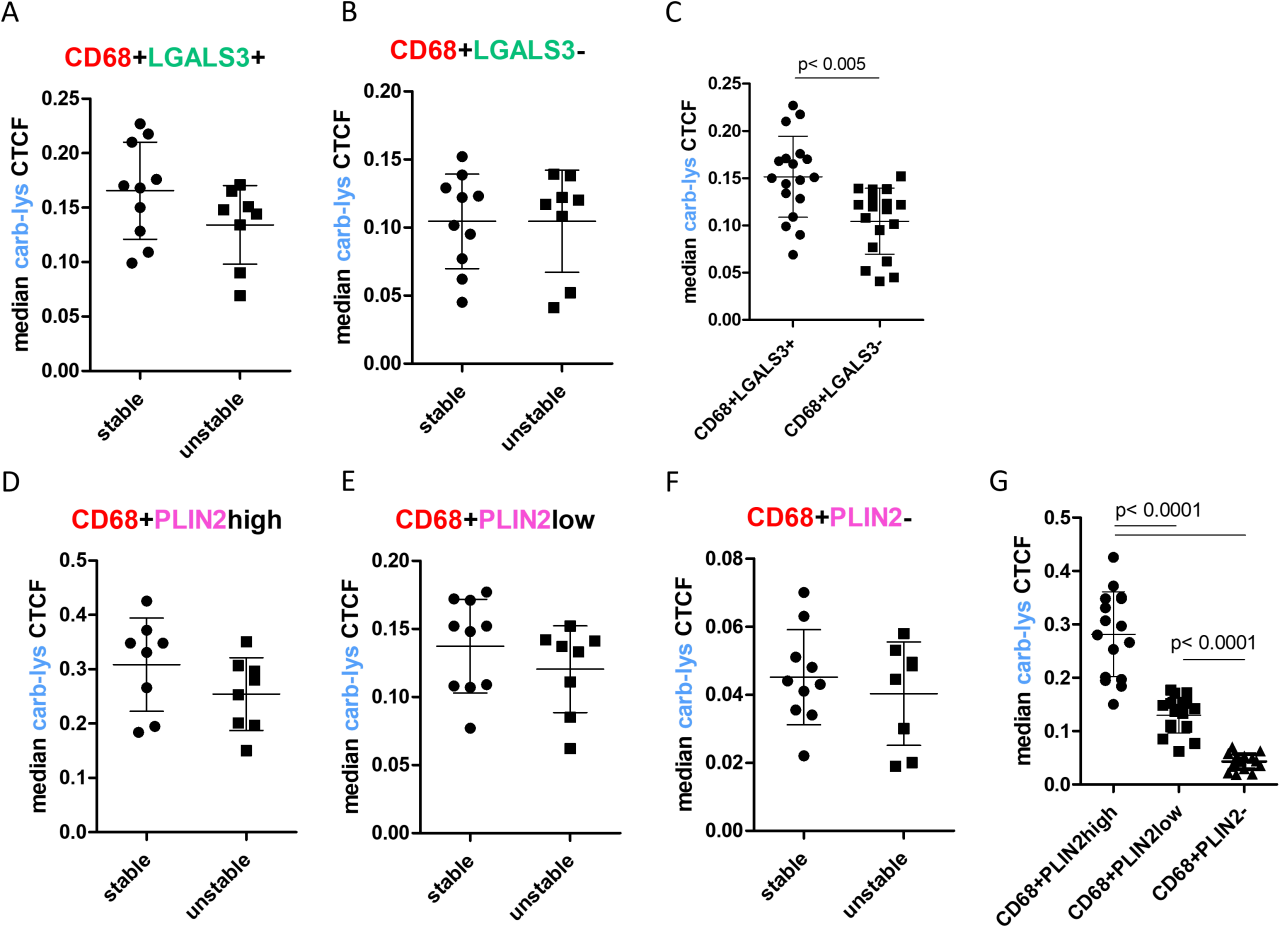
Carb-lys signal associates with macrophage foam cell markers independently of plaque stage. **(A-C)** Carb-lys median CTCF values in CD68+LGLAS3+ and CD68+LGALS3- cells in stable vs unstable plaques **(A, B)** or all plaques **(C)**. **(D-G)** Carb-lys median CTCF values in PLIN2high, PLIN2low, and PLIN2- CD68+ macrophages in stable vs unstable plaques **(D-F)** or all plaques **(G)**. Each point represents median CTCF values of the corresponding cells per plaque sample (n= 18 total).

**Figure 5:**
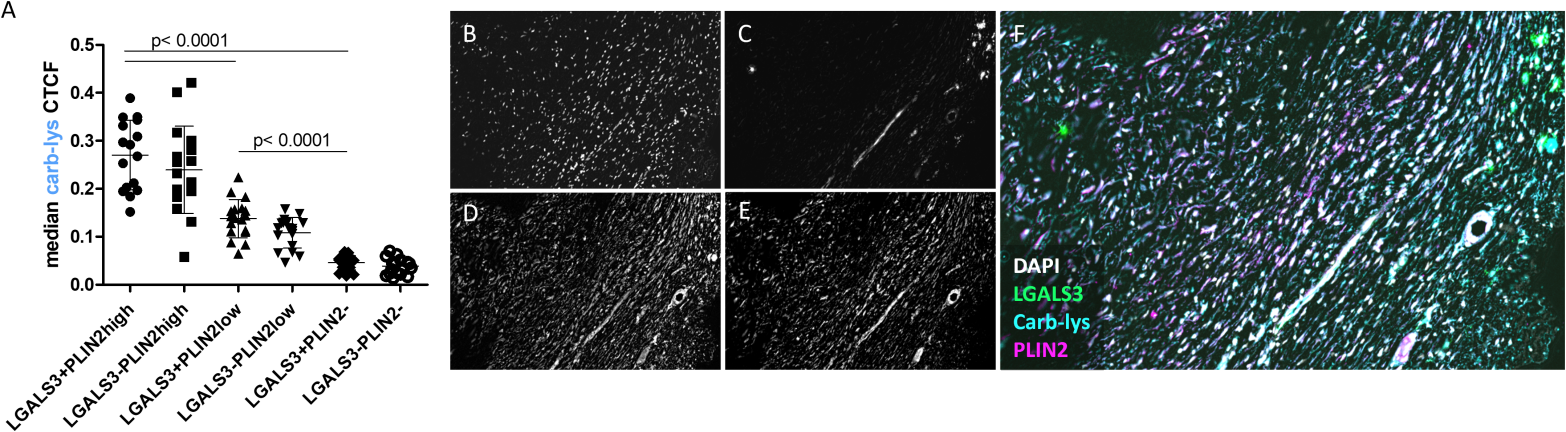
Carb-lys signal associates with PLIN2 and is independent of LGALS3 signal in CD68+ macrophages. **(A)** Carb-lys median CTCF values in CD68+ double-positive subsets. **(B, C)** Representative images of an advanced plaque sample showing co-localization of carb-lys signal with PLIN2. Representative images showing fluorescent staining of **(B)** DAPI, **(C)** LGALS3, **(D)** carb-lys, **(E)** PLIN2, and **(F)** merged channels.

Next, we compared carb-lys signal in the LGALS3^+^ vs PLIN2^+^ foam cell subsets. Irrespective of the plaque type (i.e. stable or unstable), we found a significantly higher carb-lys signal in CD68^+^ macrophages expressing LGALS3 (**Figures 4A–C**) and particularly PLIN2 (**Figures 4D–G**), underpinning our earlier observations. Interestingly, when analyzing LGALS3 and PLIN2 double-positive subsets, carb-lys signal appeared to be mainly dependent on PLIN2 rather than on LGALS3 expression (**Figures 5A–F**). It is worth noting that LGALS3 was also much less abundantly present in plaque than PLIN2 showing a lower number of positive cells (**Figure 5C**; **Supplementary Figures S4C, G, K**).

### 3.3 Carbamylated LDL uptake as main source of intracellular carbamylation signal

Previously, pro-modifying enzymes such as myeloperoxidase (MPO) highly expressed by myeloid cells and, in particular, neutrophils, under pro-inflammatory conditions in plaque (40), have been reported to be capable of catalyzing protein carbamylation (4, 5, 41). Indeed, in our study, MPO also showed a good correlation to carb-lys signal with the Pearson’s coefficient of 0.584 (**Supplementary Figures S5A–D**). However, only 7.6% of total carb-lys^+^ area was overlapping with the MPO signal, implying presence of a substantial amount of carbamylated protein in MPO-deficient areas. Additionally, carb-lys median CTCF did not correlate with the relative MPO gene expression levels in the MaasHPS cohort (ρ= 0.0795, ns) (**Supplementary Figure S5E**).

**Figure 6:**
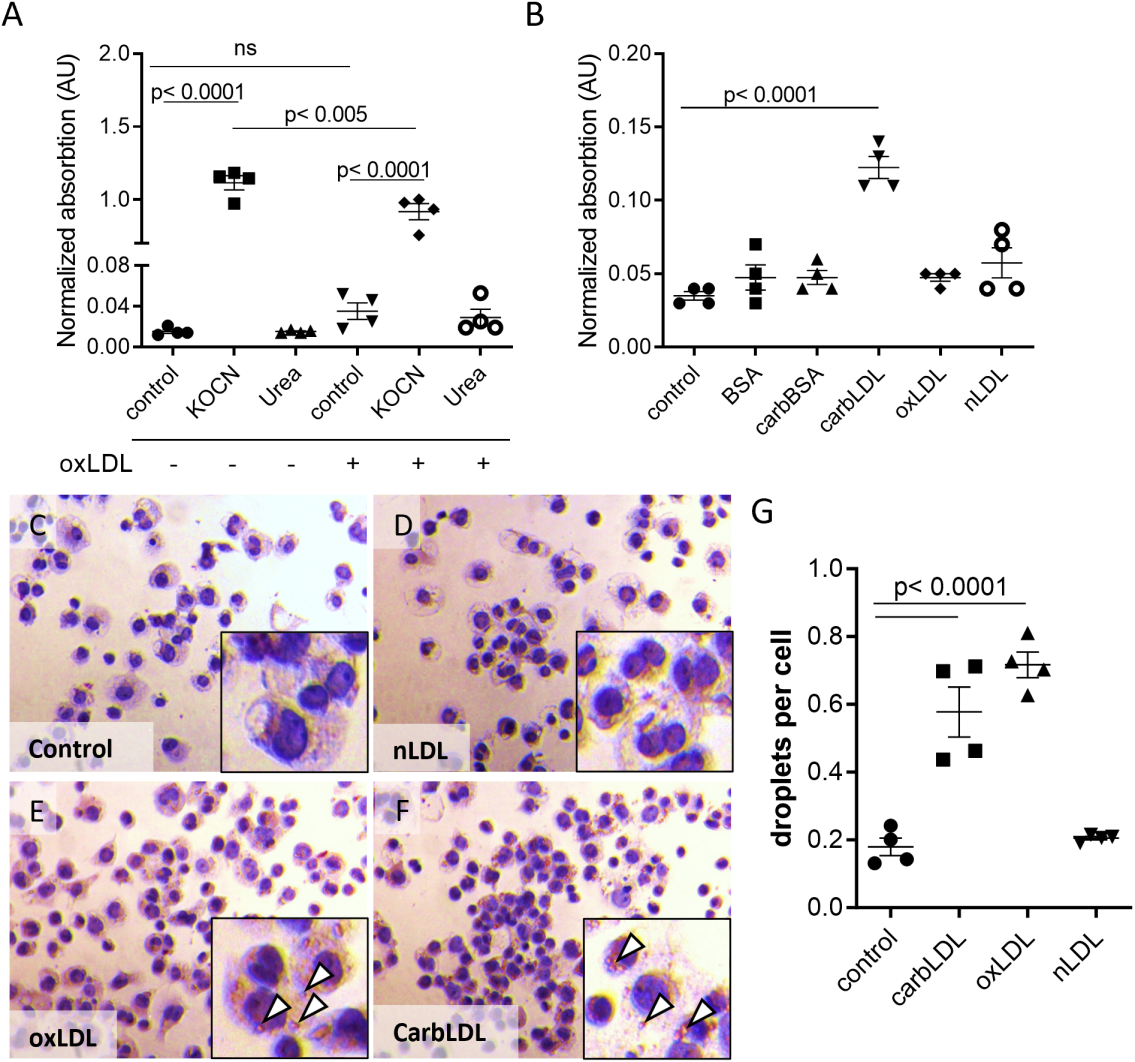
carbLDL uptake increases intracellular carbamylation and induces foam cell formation. **(A)** Carb-lys levels in native and oxLDL-induced THP-1 derived foam cells treated with 5 mM KOCN or 40 mM urea for 24 hours. **(B)** Carb-lys levels in THP-1 cells stimulated with either 20 μg/ml BSA, carbBSA, carbLDL, oxLDL, non-modified LDL (nLDL) or no treatment (control) for 24 hours. Carb-lys signal measured in cell lysates with ELISA and expressed in adsorption values normalized to total protein concentration, n= 4 per condition. **(C-F)** Representative images of Oil Red O staining showing THP-1 macrophages preliminary treated with modified or non-modified LDL (20 μg/ml) for 24 hours. Lipid droplets (white arrowheads) can be seen in the cytoplasm. **(G)** Quantification of lipid droplets in the treated cells expressed in the average number of droplets per cell. Points represent individual wells (n= 4).

The correlation of plaque carb-lys signal with kidney function decline led us to investigate whether plaque protein carbamylation could be attributed to the effects of increased urea levels in these CKD patients. To address urea exposure as a potential source of macrophage protein carbamylation, we treated THP-1 derived macrophages *in vitro* with urea and a known carbamylating agent (KOCN) as a reference and analyzed cellular carb-lys content using ELISA. As expected, KOCN induced strong protein carbamylation both in native macrophages and oxLDL-induced foam cells (**Figure 6A**) with foam cells being slightly less susceptible to carbamylation than the non-foamy macrophages. KOCN and urea treatment did not reduce cell viability (data not shown).While ELISA was unable to detect any carbamylation signal after macrophage exposure to urea (**Figure 6A**), analysis by more sensitive and specific MS/MS did reveal clear carbamylation signals for both KOCN and urea (**Supplementary Figure S6**). Next, we examined whether uptake of extracellularly formed carbamylated proteins could be a source of intracellular carbamylation signal in the plaque cells. Unlike carbamylated BSA, carbLDL did cause a strong increase in carb-lys levels, as compared to untreated or native LDL- and oxLDL- treated macrophages (**Figure 6B**).

To address if the increased lys-carb might be resulting from the intracellular accumulation of carbLDL, we tested this particle’s capacity to induce foam cell formation in comparison to oxLDL. Indeed, Oil Red O staining showed a similar degree of lipid droplet formation in THP-1 macrophages exposed for 24-hour to carbLDL versus oxLDL (**Figures 6C–G**). However, short-term (2.5-hour) uptake of carbLDL particles was lower than that of oxLDL (**Supplementary Figures S7A–C**), suggestive of slower uptake kinetics. Interestingly, confocal imaging revealed pronounced accumulation of carb-lys signal in the submembrane region of human macrophages treated for 24 hours with carbLDL (**Supplementary Figures S7D–F**). This suggests that carbLDL particles might become trapped in the early endosomal compartment. Surprisingly, we also observed pronounced nuclear carb-lys signal, which was also present in the untreated cells (data not shown).

Taken together, these data indicate that the increase in total carbamylated protein in later-stage plaques of uremic patients can be, at least partially, explained by extracellular carbLDL uptake by macrophages.

### 3.4 carbLDL impacts macrophage function, but through a different mechanism than oxLDL

**Figure 7:**
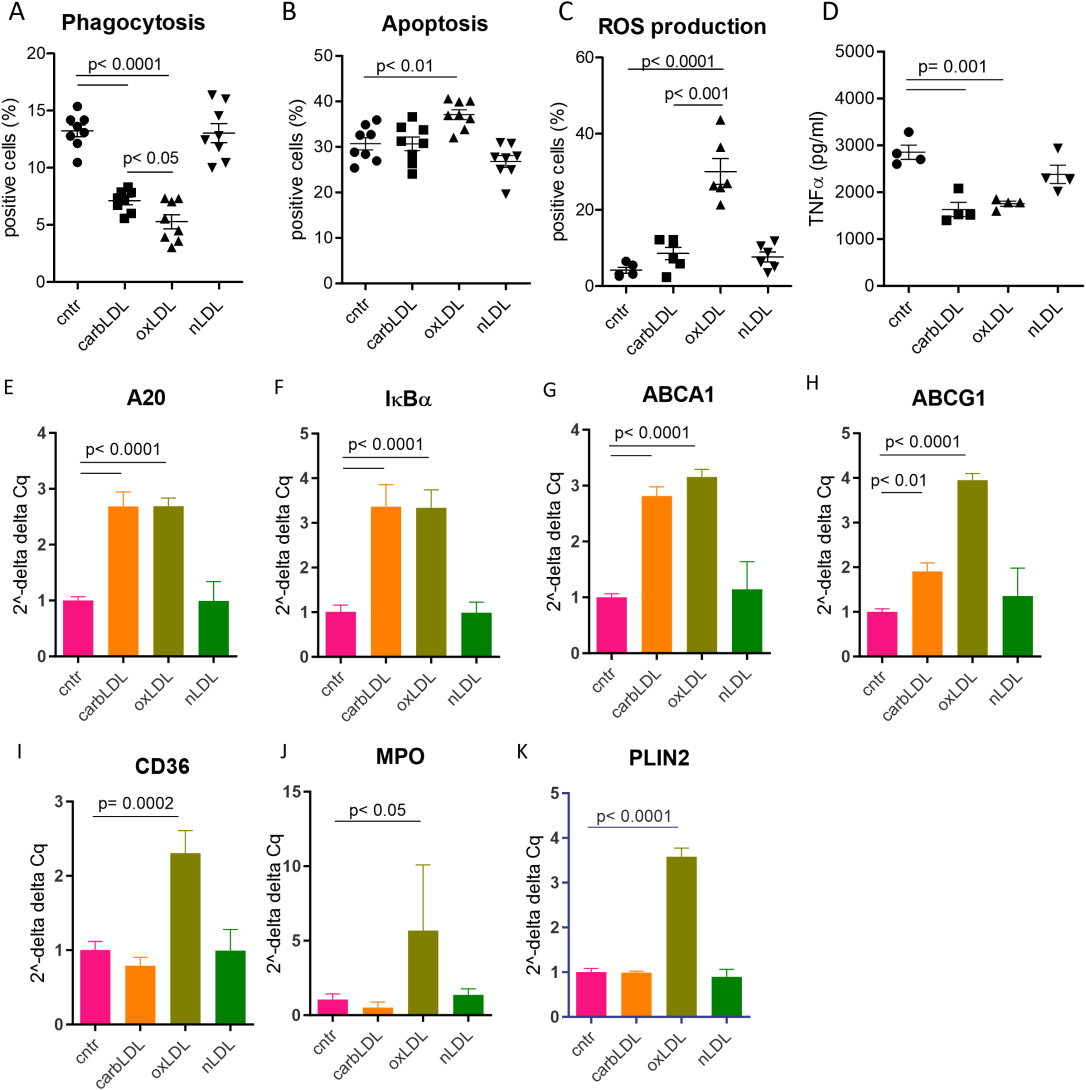
carbLDL treatment shows less pronounced functional effects on macrophages compared to oxLDL as well as differential effects on PPARγ pathway activation. **(A)** Phagocytosis levels of pHrodo zymosan particles expressed in the percentage of positive cells, n= 8. **(B)** TNFα (pg/ml of media) produced by THP-1 derived macrophages, pre - treated for 24 hours with (modified) LDL, followed by 6h LPS stimulation, n= 8. **(C)** Level of apoptosis in THP-1 derived foam cells expressed as percentage of annexin V-OG positive cells, n= 8. **(D)** ROS production by THP-1 derived foam cells in response to PMA stimulation expressed as percentage of DCFDA positive cells, n= 8. **(E-K)** qPCR results of carbLDL, oxLDL and nLDL (native) treated THP-1 cells normalized to non-treated cells and expressed in 2^–ΔΔCq^ values, n= 4.

Finally, we sought to investigate the functional impact of carbLDL uptake on macrophages. THP-1 derived macrophages were laden with modified LDLs for 24 hours and relevant functions profiled by the MacroScreen (32, 33). CarbLDL treatment significantly dampened the macrophages’ phagocytic capacity, however to a lower extent compared to oxLDL (**Figure 7A**). As shown previously (42, 43), macrophages exposure to oxLDL induced apoptosis and strongly stimulated ROS production. In contrast, however, carbLDL treatment did not increase apoptosis or ROS production (**Figures 7B, C**). Both carbLDL- and oxLDL-generated foam cells similarly reduced TNFα production response upon LPS stimulation (**Figure 7D**).

To elucidate the mechanisms by which carbLDL affects macrophage functions in comparison to oxLDL, we performed qPCR analysis of foam cells induced with these modified particles (24h exposure). A20 and IκBα expression, both measures of inflammatory NF-κB pathway activation, were induced at a similar extent by carb- and oxLDL treatment (**Figures 7E, F**), confirming previous findings (44). Cholesterol transporters ABCA1 and ABCG1 were also upregulated to comparable levels by both lipoproteins (**Figures 7G, H**). On the other hand, expression of established PPARγ reporter genes, such as CD36, MPO, and PLIN2, were induced by oxLDL while carbLDL had either no effect (PLIN2 and MPO) or even tended to downregulate their expression (CD36; **Figures 7I–K**). These data suggest that carbLDL induces pro-inflammatory signaling similar to oxLDL but is unable to spur PPARγ dependent lipid handling mechanisms.

## 4 Discussion

Carbamylation is a common non-enzymatic post-translational protein modification that accumulates in the organism with age as well as in case of systemic disorders such as kidney disease, rheumatoid arthritis, and CVD (6). However, the underlying mechanism and consequences of this modification are only poorly understood.

In this study we show that carbamylated lysine (carb-lys) signal increases in late-stage atherosclerotic plaque, particularly in patients with reduced kidney function, i.e. under uremic conditions. As previously reported, our cohort also exhibited significantly elevated serum urea levels in CKD patients. While CKD/non-CKD patients were age- and sex matched and all patients in this study were hypertensive, no significant differences were observed in any other cardiovascular risk factors (shown in **Supplementary Table S2**), except for plasma urea levels. Plaque carbamylation showed a borderline significant correlation with serum urea levels (r=0.45, p=0.083), whereas no correlation was observed with cardiovascular risk factors, including smoking. However, as mentioned, the statistical power of this study is too low to draw definitive conclusions.

Interestingly, while KOCN exposure led to an elevated carbamylation signal, ELISA analysis failed to detect any changes in overall protein carbamylation levels following *in vitro* treatment of macrophages with urea. However, using more sensitive MS/MS analyses, we identified a significant increase in the carbamylation of α-tubulin and β-actin in urea treated cells. It should be noted though that *in vitro* treatment involves short exposure (24 hours) to very high urea concentrations (albeit still within the limits observed in patients with CKD; 45), and therefore not fully reflective of the clinical condition, in which CKD patients are chronically exposed to high urea levels. Analyzing the sub-cohort of published MaasHPS study (21), we observed a significant correlation between CD68 macrophages and cellular carb-lys signal. Additionally, a strong association was found between carb-lys and PLIN2, a known lipid droplet-associated protein and (non-specific) foam cell marker in mice (24, 46), as well as with LGALS3, another foam cell marker, independently of plaque stages. CarbLDL uptake and accumulation was previously demonstrated to cause foam cell formation in macrophages through recognition by scavenger receptors CD36, lectin-like-oxidized low-density lipoprotein receptor-1 (LOX-1) and scavenger receptor A1 (SR-A1), although to a different extent than that of oxLDL, the preferred ligand for the LOX-1 and SR-A1 (14). Indeed, our *in vitro* experiments confirm carbLDL induced foam cell formation for human macrophages and, in addition, highlight that the increase of intracellular carb-lys content only occurs after carbLDL, but not after carbamylated albumin or oxLDL exposure^36^. These findings render ingested carbamylated LDL a primary source of carbamylated protein accumulation in plaque cells, particularly as MPO (another suggested source of carbamylation 4, 5, 41) showed only 7% overlap with carb-lys staining in plaque. To what extent carbamylated HDL, which has been shown to be present in atherosclerotic lesions (12), also contributes to carb-protein accumulation remains to be shown. It should be mentioned, however, that since the CKD vs non-CKD cohort samples were not studied for the foam cell abundancy, it is not known if the increase of carb-lys signal in the advanced plaques originates from the elevated infiltration of the foamy carbLDL-laden macrophages, or due to increased amount of carbamylated LDL particles per cell. In addition, urea blood levels were shown previously to correlate with LDL carbamylation (47). As tissue urea information is not available for the used cohorts, it is hard to say to what extent LDL carbamylation occurs within the plaque tissue compared to that originating from the circulation, as was shown for a hyperlipidemic CKD mouse model (47).

*In vitro*, carbLDL triggered inflammatory pathway to a similar extent as oxLDL, judging by the increased A20 and IκBα expression, both well-known negative regulators of NF-kB (48, 49). This effect could well underlie the dampened TNFα response to LPS treatment in carbLDL-laden macrophages. OxLDL was shown earlier to activate NF-κB pathway through e.g., its recognition by CD36 and Toll-like receptor 4 (TLR-4) (50, 51), as well as through induction of ROS as a result of binding to LOX-1 (52). Consistently with the above, carbLDL was also demonstrated to upregulate NF-κB through interaction with LOX-1 (44). On the other hand, carbLDL did not show ROS or apoptosis-inducing ability in our setup, which, at the first glance, contradicts the published data (8, 14, 17, 18, 53, 54). Nevertheless, this discrepancy might come from the fact that the published studies were performed in different cell types using much higher LDL concentrations. However, the exact mechanisms behind carbLDL mode of action require further investigation.

Interestingly, even though we observed a strong correlation between carb-lys and PLIN2 expression in plaque, carbLDL did not upregulate PLIN2 expression in macrophages *in vitro*, in contrast to oxLDL. Most likely, however, in the pro-oxidant plaque milieu, LDL carbamylation and oxidation occurs in parallel, and the resultant double-modified carbamylated-oxLDL, previously reported in a CKD mouse model (14), will be taken up by plaque macrophages. Thus, in plaque, most of the carbLDL signal might represent the double modified LDL, which likely harbors the PPARγ activating capacity of oxLDL, explaining the observed colocalization. However, this double modified LDL is yet to be described in the plaque.

PLIN2 was shown to protect lipid droplets from disintegration and processing by lipases (55, 56), hence the lack of PLIN2 upregulation in response to carbLDL treatment could indicate a faster lipid turnover. In addition, induced expression of the cholesterol transporters by carbLDL would favor a smooth cholesterol efflux. In that regard, carbamylation may be less pro-atherogenic than oxidation. Interestingly, after uptake by the macrophages, oxLDL was reported to get trapped in the lysosomal compartment causing cholesterol crystal formation though CD36 engagement and subsequent inflammasome activation (57, 58). Our confocal imaging experiment revealed a strong localization of carbLDL in the submembranous region of human macrophages, suggesting trapping of carbLDL at an earlier stage of endolysosomal processing. This might explain why carbLDL had lower ROS and apoptosis promoting effects than oxLDL. RNA sequencing and electron microscopy experiments of carbLDL treated cells could help clarify this hypothesis in future.

Although this study was limited in power and punctiform blood urea levels were used as surrogate marker for systemic carbamylation propensity, our study shows that carbamylation levels increase in atherosclerotic plaque of patients with impaired kidney function in a stage dependent manner; carb-protein accumulation likely reflects uptake and corrupted processing of carbamylated LDL from the local milieu, as carbLDL exposure led to foam cell formation. CarbLDL accumulation caused less severe functional alterations than oxLDL, among others, by their failure to activate PPARγ. The precise mechanism of carbLDL specific effects as well as physiological relevance of carbLDL for plaque development are yet to be described.

## Data Availability

The raw data supporting the conclusions of this article will be made available by the authors, without undue reservation.

## References

[1] Poznyak AV Nikiforov NG Markin AM Kashirskikh DA Myasoedova VA Gerasimova EV. Overview of oxLDL and its impact on cardiovascular health: focus on atherosclerosis. Front Pharmacol. (2021) 11:2248. doi: 10.3389/fphar.2020.613780 PMC7836017 33510639

[2] Kalim S Berg AH Karumanchi SA Thadhani R Allegretti AS Nigwekar S. Protein carbamylation and chronic kidney disease progression in the Chronic Renal Insufficiency Cohort Study. Nephrol Dial Transplant. (2021) 37:139–47. doi: 10.1093/ndt/gfaa347 PMC8719615 33661286

[3] Kraus LM Kraus J. Carbamoylation of amino acids and proteins in uremia. Kidney Int Suppl. (2001) 78:S102–7. doi: 10.1046/j.1523-1755.2001.59780102.x 11168993

[4] Holzer M Zangger K El-Gamal D Binder V Curcic S Konya V. Myeloperoxidase-derived chlorinating species induce protein carbamylation through decomposition of thiocyanate and urea: Novel pathways generating dysfunctional high-density lipoprotein. Antioxid Redox Signal. (2012) 17:1043–52. doi: 10.1089/ars.2011.4403 PMC3810648 22462773

[5] Wang Z Nicholls SJ Rodriguez ER Kummu O Hörkkö S Barnard J. Protein carbamylation links inflammation, smoking, uremia and atherogenesis. Nat Med. (2007) 13:1176–84. doi: 10.1038/nm1637 17828273

[6] Delanghe S Delanghe JR Speeckaert R Van Biesen W Speeckaert MM. Mechanisms and consequences of carbamoylation. Nat Rev Nephrol. (2017) 13:580–93. doi: 10.1038/nrneph.2017.103 28757635

[7] Berg AH Drechsler C Wenger J Buccafusca R Hod T Kalim S. Carbamylation of serum albumin as a risk factor for mortality in patients with kidney failure. Sci Transl Med. (2013) 5(175):175ra29. doi: 10.1126/scitranslmed.3005218 PMC3697767 23467560

[8] Speer T Owala FO Holy EW Zewinger S Frenzel FL Stähli BE. Carbamylated low-density lipoprotein induces endothelial dysfunction. Eur Heart J. (2014) 35:3021–32. doi: 10.1093/eurheartj/ehu111 24658767

[9] Jaisson S Lorimier S Ricard-Blum S Sockalingum GD Delevallée-Forte C Kegelaer G. Impact of carbamylation on type I collagen conformational structure and its ability to activate human polymorphonuclear neutrophils. Chem Biol. (2006) 13:149–59. doi: 10.1016/j.chembiol.2005.11.005 16492563

[10] Binder V Chruścicka-Smaga B Bergum B Jaisson S Gillery P Sivertsen J. Carbamylation of integrin α IIb β 3: the mechanistic link to platelet dysfunction in ESKD. J Am Soc Nephrol. (2022) 33:1841–56. doi: 10.1681/ASN.2022010013 PMC9528322 36038265

[11] Binder V Bergum B Jaisson S Gillery P Scavenius C Spriet E. Impact of fibrinogen carbamylation on fibrin clot formation and stability. Thromb Haemost. (2017) 117:899–910. doi: 10.1160/TH16-09-0704 28382370 PMC5442607

[12] Holzer M Gauster M Pfeifer T Wadsack C Fauler G Stiegler P. Protein carbamylation renders high-density lipoprotein dysfunctional. Antioxid Redox Signal. (2011) 14:2337–46. doi: 10.1089/ars.2010.3640 PMC3380531 21235354

[13] Holzer M Birner-Gruenberger R Stojakovic T El-Gamal D Binder V Wadsack C. Uremia alters HDL composition and function. J Am Soc Nephrol. (2011) 22:1631–41. doi: 10.1681/ASN.2010111144 PMC3171935 21804091

[14] Apostolov EO Ok E Burns S Nawaz S Savenka A Shah SV. Carbamylated-oxidized LDL: proatherosclerotic effects on endothelial cells and macrophages. J Atheroscler Thromb. (2013) 20:878–92. doi: 10.5551/jat.14035 PMC5345570 24067603

[15] Rajamohan A Heit B Cairns E Barra L. Citrullinated and homocitrullinated low- density lipoprotein in rheumatoid arthritis. Scand J Rheumatol. (2021) 50:343–50. doi: 10.1080/03009742.2020.1867237 33678128

[16] Asci G Basci A Shah SV Basnakian A Toz H Ozkahya M. Carbamylated low-density lipoprotein induces proliferation and increases adhesion molecule expression of human coronary artery smooth muscle cells. Nephrol (Carlton). (2008) 13:480–6. doi: 10.1111/j.1440-1797.2008.00948.x 18518940

[17] Ok E Basnakian AG Apostolov EO Barri YM Shah SV. Carbamylated low-density lipoprotein induces death of endothelial cells: a link to atherosclerosis in patients with kidney disease. Kidney Int. (2005) 68:173–8. doi: 10.1111/j.1523-1755.2005.00391.x 15954906

[18] Apostolov EO Ray D Alobuia WM Mikhailova MV Wang X Basnakian AG. Endonuclease G mediates endothelial cell death induced by carbamylated LDL. Am J Physiol Heart Circ Physiol. (2011) 300:1997–2004. doi: 10.1152/ajpheart.01311.2010 PMC3119093 21460199

[19] Apostolov EO Basnakian AG Yin X Ok E Shah SV. Modified LDLs induce proliferation-mediated death of human vascular endothelial cells through MAPK pathway. Am J Physiol Heart Circ Physiol. (2007) 292:1836–46. doi: 10.1152/ajpheart.01079.2006 17158646

[20] El-Gamal D Rao SP Holzer M Hallström S Haybaeck J Gauster M. The urea decomposition product cyanate promotes endothelial dysfunction. Kidney Int. (2014) 86:923–31. doi: 10.1038/ki.2014.218 PMC4216595 24940796

[21] Jin H Goossens P Juhasz P Eijgelaar W Manca M Karel JMH. Integrative multiomics analysis of human atherosclerosis reveals a serum response factor-driven network associated with intraplaque hemorrhage. Clin Transl Med. (2021) 11:11. doi: 10.1002/ctm2.v11.6 PMC8236116 34185408

[22] Virmani R Kolodgie FD Burke AP Farb A Schwartz SM. Lessons from sudden coronary death. Arterioscler Thromb Vasc Biol. (2000) 20:1262–75. doi: 10.1161/01.ATV.20.5.1262 10807742

[23] Levey A Stevens L Schmid C Zhang Y Castro A III Feldman H. A new equation to estimate glomerular filtration rate. Ann Intern Med. (2009) 150:604–12. doi: 10.7326/0003-4819-150-9-200905050-00006 PMC2763564 19414839

[24] Goossens P Lu C Cao J Gijbels MJ Karel JMH Wijnands E. Integrating multiplex immunofluorescent and mass spectrometry imaging to map myeloid heterogeneity in its metabolic and cellular context. Cell Metab. (2022) 34:1214–1225.e6. doi: 10.1016/j.cmet.2022.06.012 35858629

[25] Schindelin J Arganda-Carreras I Frise E Kaynig V Longair M Pietzsch T. Fiji: an open-source platform for biological-image analysis. Nat Methods. (2012) 9:7 9, 676–682. doi: 10.1038/nmeth.2019 PMC3855844 22743772

[26] Bolte S Cordelières FP. A guided tour into subcellular colocalization analysis in light microscopy. J Microsc. (2006) 224:213–32. doi: 10.1111/j.1365-2818.2006.01706.x 17210054

[27] Guillot A Kohlhepp MS Bruneau A Heymann F Tacke F. Deciphering the immune microenvironment on A single archival formalin-fixed paraffin-embedded tissue section by an immediately implementable multiplex fluorescence immunostaining protocol. Cancers. (2020) 12:2449. doi: 10.3390/cancers12092449 32872334 PMC7565194

[28] Du Z Lin JR Rashid R Maliga Z Wang S Aster JC. Qualifying antibodies for image-based immune profiling and multiplexed tissue imaging. Nat Protoc. (2019) 10:2900–30. doi: 10.1038/s41596-019-0206-y PMC6959005 31534232

[29] Guillot A Winkler M Silva Afonso M Aggarwal A Lopez D Berger H. Mapping the hepatic immune landscape identifies monocytic macrophages as key drivers of steatohepatitis and cholangiopathy progression. Hepatology. (2023) 78:150–66. doi: 10.1097/HEP.0000000000000270 36630995

[30] McCloy RA Rogers S Caldon CE Lorca T Castro A Burgess A. Partial inhibition of Cdk1 in G2 phase overrides the SAC and decouples mitotic events. Cell Cycle. (2014) 13:1400–12. doi: 10.4161/cc.28401 PMC4050138 24626186

[31] Redgrave TG Roberts DCK West CE. Separation of plasma lipoproteins by density-gradient ultracentrifugation. Anal Biochem. (1975) 65:42–9. doi: 10.1016/0003-2697(75)90488-1 165752

[32] Fontaine MAC Jin H Gagliardi M Rousch M Wijnands E Stoll M. Blood milieu in acute myocardial infarction reprograms human macrophages for trauma repair. Adv Sci. (2023) 10(5):e2203053. doi: 10.1002/advs.202203053 PMC9929255 36526599

[33] Ruder AV Temmerman L van Dommelen JMA Nagenborg J Lu C Sluimer JC. Culture density influences the functional phenotype of human macrophages. Front Immunol. (2023) 14:1078591. doi: 10.3389/fimmu.2023.1078591 36969194 PMC10036771

[34] Stirling DR Swain-Bowden MJ Lucas AM Carpenter AE Cimini BA Goodman A. CellProfiler 4: improvements in speed, utility and usability. BMC Bioinf. (2021) 22:1–11. doi: 10.1186/s12859-021-04344-9 PMC8431850 34507520

[35] Vermes I Haanen C Steffens-Nakken H Reutellingsperger C. A novel assay for apoptosis Flow cytometric detection of phosphatidylserine expression on early apoptotic cells using fluorescein labelled Annexin V. J Immunol Methods. (1995) 184:39–51. doi: 10.1016/0022-1759(95)00072-I 7622868

[36] Kork F Jankowski J Goswami A Weis J Brook G Yamoah A. Golgin A4 in CSF and granulovacuolar degenerations of patients with Alzheimer disease. Neurology. (2018) 91:E1799–808. doi: 10.1212/WNL.0000000000006457 30305446

[37] Madiyal A Ajila V Babu SG Hegde S Kumari S Madi M. Status of thiocyanate levels in the serum and saliva of non-smokers, ex-smokers and smokers. Afr Health Sci. (2018) 18:727. doi: 10.4314/ahs.v18i3.31 30603006 PMC6307002

[38] Ospelt C Bang H Feist E Camici G Keller S Detert J. Carbamylation of vimentin is inducible by smoking and represents an independent autoantigen in rheumatoid arthritis. Ann Rheum Dis. (2017) 76:1176–83. doi: 10.1136/annrheumdis-2016-210059 PMC5530349 28183721

[39] Apostolov EO Shah SV Ray D Basnakian AG. Scavenger receptors of endothelial cells mediate the uptake and cellular proatherogenic effects of carbamylated LDL. Arterioscler Thromb Vasc Biol. (2009) 29:1622–30. doi: 10.1161/ATVBAHA.109.189795 PMC5075391 19696406

[40] Teng N Maghzal GJ Talib J Rashid I Lau AK Stocker R. The roles of myeloperoxidase in coronary artery disease and its potential implication in plaque rupture. Redox Rep. (2017) 22:51. doi: 10.1080/13510002.2016.1256119 27884085 PMC6837458

[41] Delporte C Boudjeltia KZ Furtmüller PG Maki RA Dieu M Noyon C. Myeloperoxidase-catalyzed oxidation of cyanide to cyanate: A potential carbamylation route involved in the formation of atherosclerotic plaques? J Biol Chem. (2018) 293:6374–86. doi: 10.1074/jbc.M117.801076 PMC5925816 29496995

[42] Lara-Guzmán OJ Gil-Izquierdo Á Medina S Osorio E Álvarez-Quintero R Zuluaga N. Oxidized LDL triggers changes in oxidative stress and inflammatory biomarkers in human macrophages. Redox Biol. (2018) 15:1–11. doi: 10.1016/j.redox.2017.11.017 29195136 PMC5723280

[43] Wintergerst ES Jelk J Rahner C Asmis R. Apoptosis induced by oxidized low density lipoprotein in human monocyte-derived macrophages involves CD36 and activation of caspase-3. Eur J Biochem. (2000) 267:6050–9. doi: 10.1046/j.1432-1327.2000.01682.x 10998066

[44] Holy EW Akhmedov A Speer T Camici GG Zewinger S Bonetti N. Carbamylated low-density lipoproteins induce a prothrombotic state via LOX-1: impact on arterial thrombus formation *in vivo* . J Am Coll Cardiol. (2016) 68:1664–76. doi: 10.1016/j.jacc.2016.07.755 27712780

[45] Vanholder R Gryp T Glorieux G. Urea and chronic kidney disease: the comeback of the century? (in uraemia research). Nephrol Dialysis Transplant. (2018) 33:4–12. doi: 10.1093/ndt/gfx039 28407121

[46] Itabe H Yamaguchi T Nimura S Sasabe N. Perilipins: a diversity of intracellular lipid droplet proteins. Lipids Health Dis. (2017) 16(1):83. doi: 10.1186/s12944-017-0473-y 28454542 PMC5410086

[47] Apostolov EO Ray D Savenka AV Shah SV Basnakian AG. Chronic uremia stimulates LDL carbamylation and atherosclerosis. J Am Soc Nephrol. (2010) 21:1852–7. doi: 10.1681/ASN.2010040365 PMC3014000 20947625

[48] Martens A van Loo G. A20 at the crossroads of cell death, inflammation, and autoimmunity. Cold Spring Harb Perspect Biol. (2020) 12:a036418. doi: 10.1101/cshperspect.a036418 31427375 PMC6942121

[49] Wu Y He X Huang N Yu J Shao B. A20: a master regulator of arthritis. Arthritis Res Ther. (2020) 22:1 22, 1–15. doi: 10.1186/s13075-020-02281-1 PMC7504854 32958016

[50] Janabi M Yamashita S Hirano KI Sakai N Hiraoka H Matsumoto K. Oxidized LDL-induced NF-kappa B activation and subsequent expression of proinflammatory genes are defective in monocyte-derived macrophages from CD36-deficient patients. Arterioscler Thromb Vasc Biol. (2000) 20:1953–60. doi: 10.1161/01.ATV.20.8.1953 10938017

[51] Kawai T Akira S. Signaling to NF-κB by toll-like receptors. Trends Mol Med. (2007) 13:460–9. doi: 10.1016/j.molmed.2007.09.002 18029230

[52] Robbesyn F Salvayre R Negre-Salvayre A. Dual role of oxidized LDL on the NF-kappaB signaling pathway. Free Radic Res. (2004) 38:541–51. doi: 10.1080/10715760410001665244 15346645

[53] Carracedo J Merino A Briceño C Soriano S Buendía P Calleros L. Carbamylated low-density lipoprotein induces oxidative stress and accelerated senescence in human endothelial progenitor cells. FASEB J. (2011) 25:1314–22. doi: 10.1096/fj.10-173377 21228221

[54] Son JN Lho Y Shin S Kwon SH Moon KC Ha E. Carbamylated low-density lipoprotein increases reactive oxygen species (ROS) and apoptosis via lectin-like oxidized LDL receptor (LOX-1) mediated pathway in human umbilical vein endothelial cells. Int J Cardiol. (2011) 146:428–30. doi: 10.1016/j.ijcard.2010.10.098 21094547

[55] Larigauderie G Furman C Jaye M Lasselin C Copin C Fruchart JC. Adipophilin enhances lipid accumulation and prevents lipid efflux from THP-1 macrophages: potential role in atherogenesis. Arterioscler Thromb Vasc Biol. (2004) 24:504–10. doi: 10.1161/01.ATV.0000115638.27381.97 14707038

[56] Xu S Zou F Diao Z Zhang S Deng Y Zhu X. Perilipin 2 and lipid droplets provide reciprocal stabilization. Biophys Rep. (2019) 5:3 5, 145–160. doi: 10.1007/s41048-019-0091-5

[57] Bieghs V Walenbergh SMA Hendrikx T van Gorp PJ Verheyen F Olde Damink SW. Trapping of oxidized LDL in lysosomes of Kupffer cells is a trigger for hepatic inflammation. Liver Int. (2013) 33:1056. doi: 10.1111/liv.2013.33.issue-7 23617943 PMC4040540

[58] Sheedy FJ Grebe A Rayner KJ Kalantari P Ramkhelawon B Carpenter SB. CD36 coordinates NLRP3 inflammasome activation by facilitating the intracellular nucleation from soluble to particulate ligands in sterile inflammation. Nat Immunol. (2013) 14:812. doi: 10.1038/ni.2639 23812099 PMC3720827

