# Correction: Protein carbamylation in atherosclerotic plaques correlates with uremia and disease progression, localizing predominantly to foam cells

**DOI:** 10.3389/fimmu.2025.1637511

**Published:** 2025-06-23

**Authors:** Valeria Saar-Kovrov, Aleksandra Pawlowska, Adrien Guillot, Marion J. J. Gijbels, Judith C. Sluimer, Lieve Temmerman, Pieter Goossens, Barend M. E. Mees, Frank Tacke, Vera Jankowski, Joachim Jankowski, Marjo M. P. C. Donners, Erik A. L. Biessen

**Affiliations:** ^1^ Department of Pathology, Cardiovascular Research Institute Maastricht, Maastricht University Medical Center, Maastricht, Netherlands; ^2^ Department of Hepatology and Gastroenterology, Charité - Universitätsmedizin Berlin, Campus Virchow-Klinikum and Campus Charité Mitte, Berlin, Germany; ^3^ School for Oncology and Developmental Biology (GROW), Maastricht University Medical Center, Maastricht, Netherlands; ^4^ Department of Medical Biochemistry, Experimental Vascular Biology, Amsterdam University Medical Centers, University of Amsterdam, Amsterdam, Netherlands; ^5^ Centre for Cardiovascular Science, University of Edinburgh, Edinburgh, United Kingdom; ^6^ Department of Surgery, Maastricht University Medical Center (UMC)+, Maastricht, Netherlands; ^7^ Institute for Molecular Cardiovascular Research (IMCAR), Rheinisch-Westfälische Technische Hochschule (RWTH) Aachen University, Aachen, Germany

**Keywords:** atherosclerosis, macrophages, foam cells, carbamylation, kidney disease

In the published article, there was an error in the **Funding** statement. The funding mentioned “This project has received funding from the European Union’s Horizon 2020 research and innovation programme under the Marie Skłodowska-Curie grant agreement No 764474 (CaReSyAn).” was incomplete. The correct **Funding** statement appears below.

“This project has received funding from the European Union’s Horizon 2020 research and innovation programme under the Marie Skłodowska-Curie grant agreement No 764474 (CaReSyAn). VJankowski and JJankowski are funded by the German Research Foundation (DFG) through the Transregional Collaborative Research Center (TRR 219; Project ID 322900939), (subproject S-03), (INST 948/4S-1); CRU 5011 project number 445703531, Cost-Action CA 21165, IZKF Multiorgan complexity in Friedreich Ataxia, and Phase Transition in Disease 1-1), ERA-PerMed (ERA-PERMED2022-202-KidneySign).”

The original article has been updated.

