# Supplementary material for: Protein carbamylation in atherosclerotic plaques correlates with uremia and disease progression, localizing predominantly to foam cells
Source: Front Immunol. 2025 May 28;16:1532250. doi: 10.3389/fimmu.2025.1532250 (PMC12151832; doi:10.3389/fimmu.2025.1532250)
Supplement: Supplementary file 1 [file DataSheet1.docx]

Supplementary Material

1. **Supplementary Tables**

**Table S1. Carotid artery atherosclerotic plaque types cohort**

| sample | sex | age | sample | sex | age | sample | sex | age |
| --- | --- | --- | --- | --- | --- | --- | --- | --- |
| PIT1 | M | 76 | **FCA1** | M | 72 | **IPH1** | M | 71 |
| PIT2 | M | 77 | **FCA2** | M | 70 | **IPH2** | M | 72 |
| PIT3 | F | 61 | **FCA3** | F | 75 | **IPH3** | M | 71 |
| PIT4 | M | 66 | **FCA4** | M | 58 | **IPH4** | M | 71 |
| PIT5 | M | 53 | **FCA5** | F | 59 | **IPH5** | M | 68 |
| PIT6 | M | 74 | **FCA6** | M | 70 | **IPH6** | M | 75 |
| PIT7 | M | 70 | **FCA7** | M | 66 | **IPH7** | M | 82 |
| PIT8 | M | 71 | **FCA8** | M | 79 | **IPH8** | F | 54 |
|  |  |  | **FCA9** | M | 56 | **IPH9** | M | 67 |

PIT - pathological intima thickening (68.5±8.2 years), FCA - fibrotic cap atheroma (67.2±8.0 years, p= ns vs PIT) and IPH - lesions with intraplaque haemorrhage (70.1±7.5 years, p= ns vs PIT).

**Table S2. Carotid artery atherosclerotic plaque CKD vs non-CKD cohort**

| **n** | **status** | **Plaque samples** | | | **age** | **sex** | **BMI** | **carotid stenosis (%)** | **smoker** |
| --- | --- | --- | --- | --- | --- | --- | --- | --- | --- |
|  |  | **PIT** | **FCA** | **IPH** |  |  |  |  |  |
| 1 | CKD | yes | yes | yes | 75 | Male | 24 | 74% | no |
| 2 | CKD | yes | - | yes | 85 | Male | 26 | NA | NA |
| 3 | CKD | yes | - | yes | 87 | Male | 24 | 70% | yes |
| 4 | CKD | yes | yes | yes | 84 | Male | 26 | 70% | yes |
| 5 | CKD | yes | yes | yes | 71 | Male | 29 | 85% | yes |
| 6 | CKD | yes | yes | yes | 87 | Female | 29 | NA | no |
| 7 | CKD | yes | - | yes | 80 | Female | 26 | 70% | yes |
| 8 | CKD | yes | - | yes | 76 | Female | 23 | 72% | yes |
| 9 | CKD | yes | yes | yes | 82 | Male | 26 | 69% | yes |
| 10 | CKD | yes | - | yes | 74 | Male | 23 | 80% | yes |
|  | Mean ± SD |  |  |  | 80.1±5.8 |  | 25.6±2.2 | 73.8±5.8 |  |
| 11 | non-CKD | yes | - | yes | 76 | Male | 21 | 80% | yes |
| 12 | non-CKD | yes | yes | yes | 73 | Male | 22 | NA | NA |
| 13 | non-CKD | yes | - | yes | 71 | Male | 27 | 70% | yes |
| 14 | non-CKD | yes | - | yes | 73 | Female | 25 | NA | yes |
| 15 | non-CKD | yes | yes | - | 83 | Male | 24 | 78% | yes |
| 16 | non-CKD | yes | - | yes | 80 | Male | 32 | 80% | yes |
| 17 | non-CKD | yes | - | yes | 82 | Female | 23 | 73% | no |
| 18 | non-CKD | yes | yes | yes | 75 | Female | 30 | 90% | yes |
| 19 | non-CKD | yes | yes | - | 80 | Male | 25 | 67% | yes |
|  | Mean ± SD |  |  |  | 77.0±4.4 |  | 25.4±3.6 | 76.9±7.7 |  |

PIT - pathological intima thickening, TkFCA – thick fibrotic cap atheroma and IPH - lesions with intraplaque haemorrhage.

| **n** | **status** | **eGFR** | **Urea (mmol/L)** | **cholesterol level (mmol/L)** | **Triglycerides**  **(mmol/L)** | **SBP (mmHg)** | **DBP (mmHg)** |
| --- | --- | --- | --- | --- | --- | --- | --- |
| 1 | CKD | 47 | 5.8 | 4.6 | 3.61 | 130 | 70 |
| 2 | CKD | 53 | 9.1 | 2.4 | 1.3 | 150 | 72 |
| 3 | CKD | 44.7 | 8.3 | NA | NA | 140 | 70 |
| 4 | CKD | 16 | 28.5 | 4.4 | 2.94 | 148 | 65 |
| 5 | CKD | 36.8 | 9.8 | 4.8 | 3.38 | 176 | 80 |
| 6 | CKD | 52.5 | 8.8 | 6.2 | 3.38 | 128 | 75 |
| 7 | CKD | 22.7 | 26.3 | NA | NA | 146 | 66 |
| 8 | CKD | 53 | NA | 3.7 | 2.32 | 135 | 74 |
| 9 | CKD | 47.3 | 6.9 | 4.7 | 1.17 | 150 | 77 |
| 10 | CKD | 53 | 7.8 | NA | NA | 169 | 70 |
|  | Mean ± SD | 42.6±13.4  ** | 12.34±8.6  *** | 4.4±1.1 | 2.6±1.0 | 147±15 | 72±5 |
| 11 | non-CKD | 63.2 | 4.7 | NA | NA | 160 | 74 |
| 12 | non-CKD | 65 | 4.9 | NA | NA | 180 | 90 |
| 13 | non-CKD | 80 | 4.7 | 3.8 | 1.32 | 126 | 59 |
| 14 | non-CKD | 77 | 2.7 | 5.9 | 2.6 | 116 | 72 |
| 15 | non-CKD | 66.1 | 4.2 | 5.9 | 3.26 | 150 | 80 |
| 16 | non-CKD | 61 | 6.4 | 5.3 | 1.49 | 137 | 81 |
| 17 | non-CKD | 61 | 4.9 | 4.6 | 2.83 | 165 | 79 |
| 18 | non-CKD | 79.2 | 8.2 | 3.5 | 1.47 | 134 | 69 |
| 19 | non-CKD | 85.8 | 6.9 | 3.9 | 0.74 | 123 | 60 |
|  | Mean ± SD | 70.9±9.5 | 5.3±1.6 | 4.7±1.0 | 1.9±0.9 | 143±22 | 74±10 |

** P<0.01; *** P<0.001

**Table S3. Carotid artery atherosclerotic plaque sub-cohort cohort**

| **n** | **ST/UNS** | **classification** | **Patient Sample number as in Jin et al.**1 | **Patient Pairs as in Jin. et al.**1 | **Age** | **Gender** |
| --- | --- | --- | --- | --- | --- | --- |
| 1 | Stable | TkFCA | 17 | 8 | 72 | M |
| 2 | Stable | PIT | 20 | 9 | 80 | M |
| 3 | Stable | TkFCA | 21 | 10 | 70 | M |
| 4 | Stable | TkFCA | 29 | 13 | 69 | M |
| 5 | Stable | TkFCA | 38 | 15 | 73 | M |
| 6 | Stable | TkFCA | 40 | 16 | 76 | M |
| 7 | Stable | TkFCA | 41 | 17 | 67 | M |
| 8 | Stable | TkFCA | 41 | 17 | 67 | M |
| 9 | Stable | TnFCA | 43 | 18 | 66 | M |
| 10 | Stable | TkFCA | 52 | 22 | 83 | M |
| 1 | Unstable | Ruptured | 32 | 14 | 76 | M |
| 2 | Unstable | Ruptured | 39 | 16 | 76 | M |
| 3 | Unstable | IPH | 44 | 18 | 66 | M |
| 4 | Unstable | IPH | 45 | 19 | 64 | M |
| 5 | Unstable | IPH | 46 | 19 | 64 | M |
| 6 | Unstable | IPH | 48 | 20 | 82 | M |
| 7 | Unstable | IPH | 51 | 22 | 83 | M |
| 8 | Unstable | IPH | 51 | 22 | 83 | M |

PIT - pathological intima thickening, TkFCA – thick fibrotic cap atheroma and IPH - lesions with intraplaque haemorrhage. Age: 72.3±5.8 vs 74.3±5.9, ns, stable vs unstable, respectively.

**Table S4. Used cell classification thresholds.**

| **Channel** | **Threshold Skew (should be below:)** | **Threshold CTCF (should be above:)** | **High threshold CTCF** |
| --- | --- | --- | --- |
| CD68 | 4.5 | 0.03 | - |
| LGALS3 | 2.5 | 0.03 | - |
| Carb-lys | 2.5 | 0.05 | - |
| PLIN2 | 3.0 | 0.03 | 0.200 |

CTCF – correlated total cell fluorescence.

1. **Supplementary Figures**

**
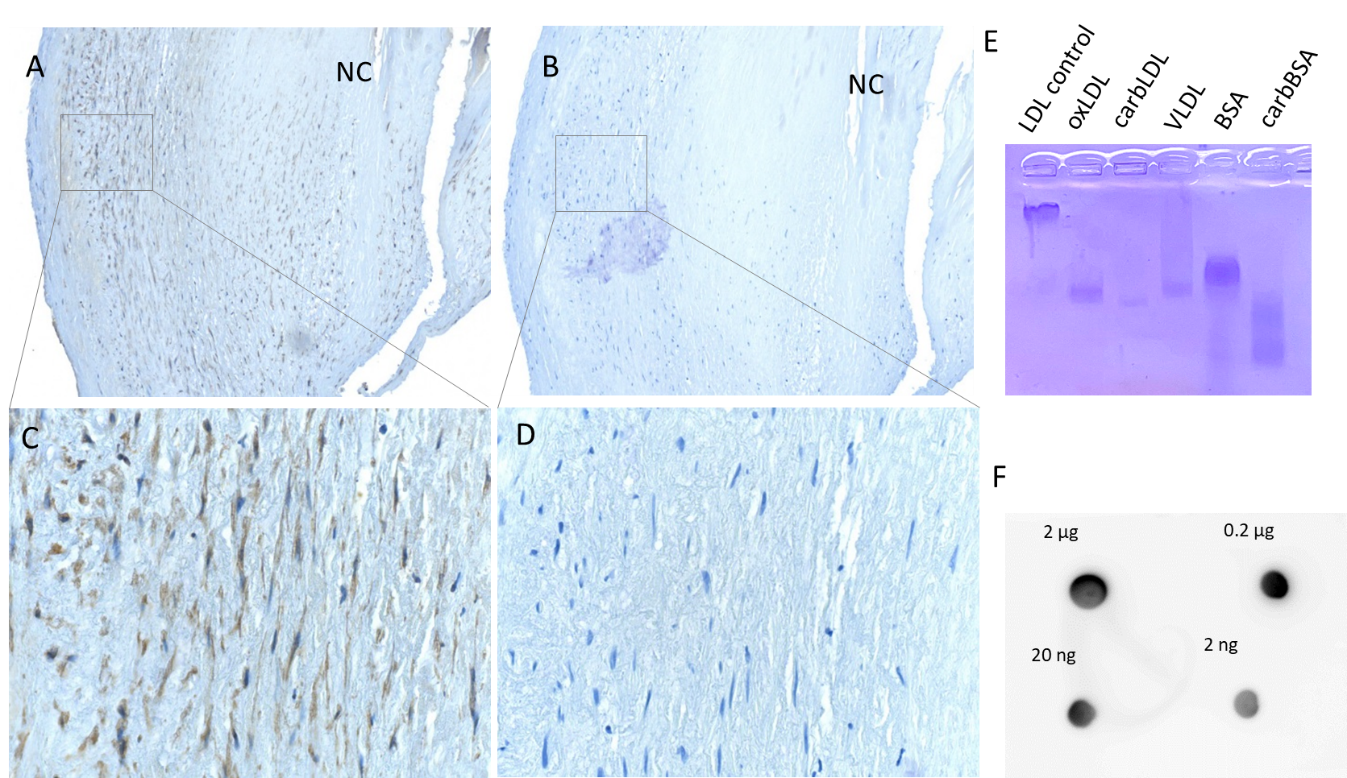
**

**Figure S1. Validation of anti-carb-lys antibody and *in vitro* carbamylation.** **A**-**D**) Immunostaining of carb-lys in an advanced plaque sample showing carb-lys positive brown signal (**A**, **C**) and no staining when the antibody was preliminary pre-adsorbed with carb-BSA (**B**, **C**). Objectives x10 (top) and x40 (bottom). **E**) Coomassie blue stained agarose gel showing non-modified and modified LDL and BSA, as indicated**. F**) Dot blot against different amount of carbamylated BSA (2 - 0.002 µg). NC – necrotic core.

**
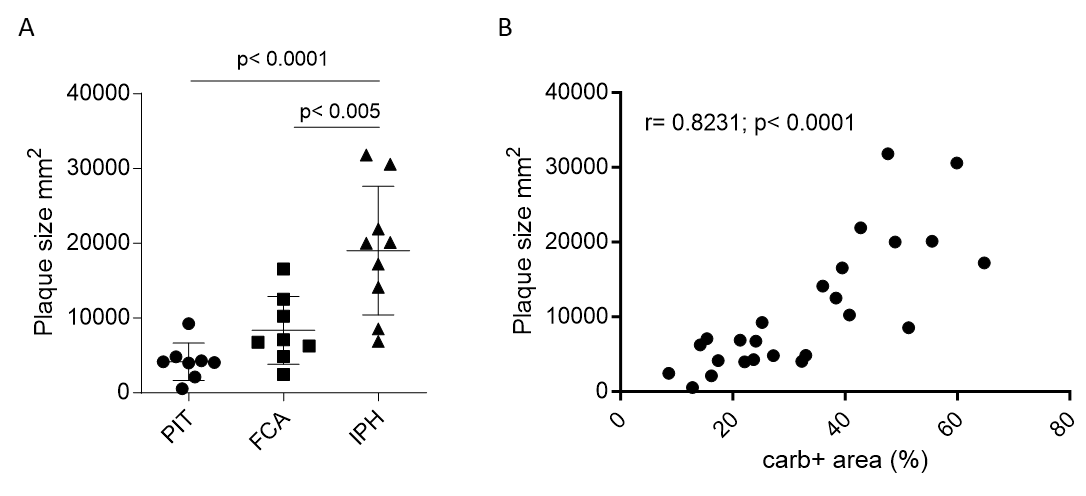
****Figure S2. Carb-lys percentage of positive area correlates with the plaque area.** **A**) Total plaque area. PIT – pathological intima thickening (early plaque stage); FCA - fibrous cap atheroma (advanced stable plaque); IPH - intraplaque hemorrhage lesions (advanced ruptured plaque). **B**) Correlation between percentage of carb-lys positive area to the total plaque area.

**
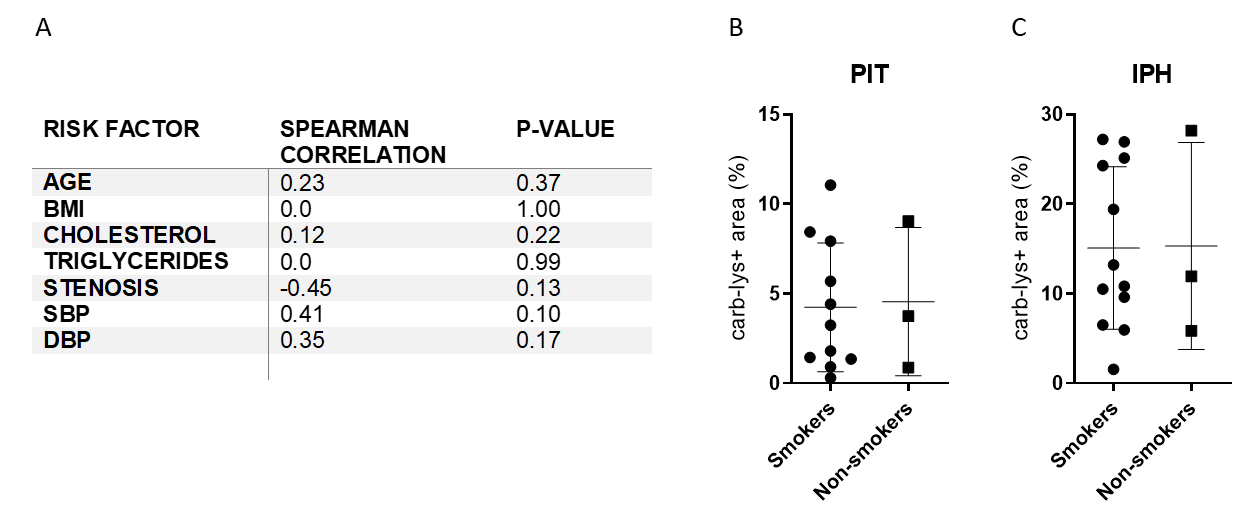
**

**Figure S3. Carb-lys positive signal percentage in plaques does not correlate with most CVD risk factors and does not differ between smoking and non-smoking patients.** **A**) Correlation of Carb-lys+ area with CVD risk factors. BMI=body mass index, SBP= systolic blood pressure, DBP= diastolic blood pressure. **B**) Carb-lys+ area percentage in PIT and **C**) IPH plaques of CKD vs non-CKD patient cohort.

**
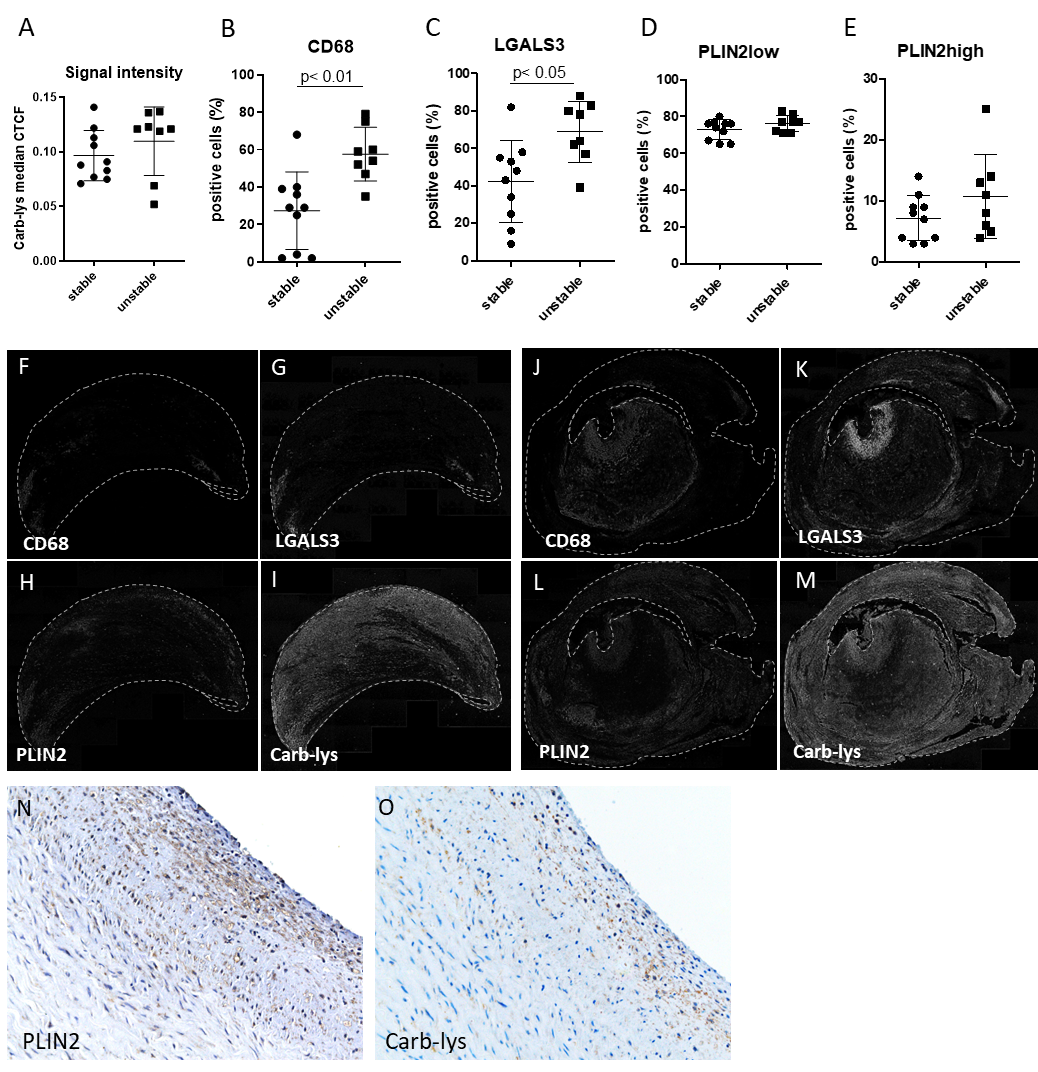
**

**Figure S4. Carb-lys and macrophage markers signal in MaasHPS sub-cohort**. **A**) Carb-lys median CTCF values in the cohort groups. **B**-**E**) Percentage of CD68, LGALS3 and PLIN2 positive cells in the cohort groups. For classifying PLIN2low a threshold of CTCF> 0.03 was used, and for PLIN2high – CTCF> 0.200. **F**-**M**) Representative images showing single channels (CD68, LGALS3, PLIN2, and carb-lys) of sequential immunostaining of stable (**F**-**I**) and unstable (**J**-**M**) plaques. **N**-**O**) representative images of adjacent plaque tissue slides stained with anti-carb-lys (**N**) or anti-PLIN2 (**O**) antibodies and visualized with DAB under x20 objective.

**
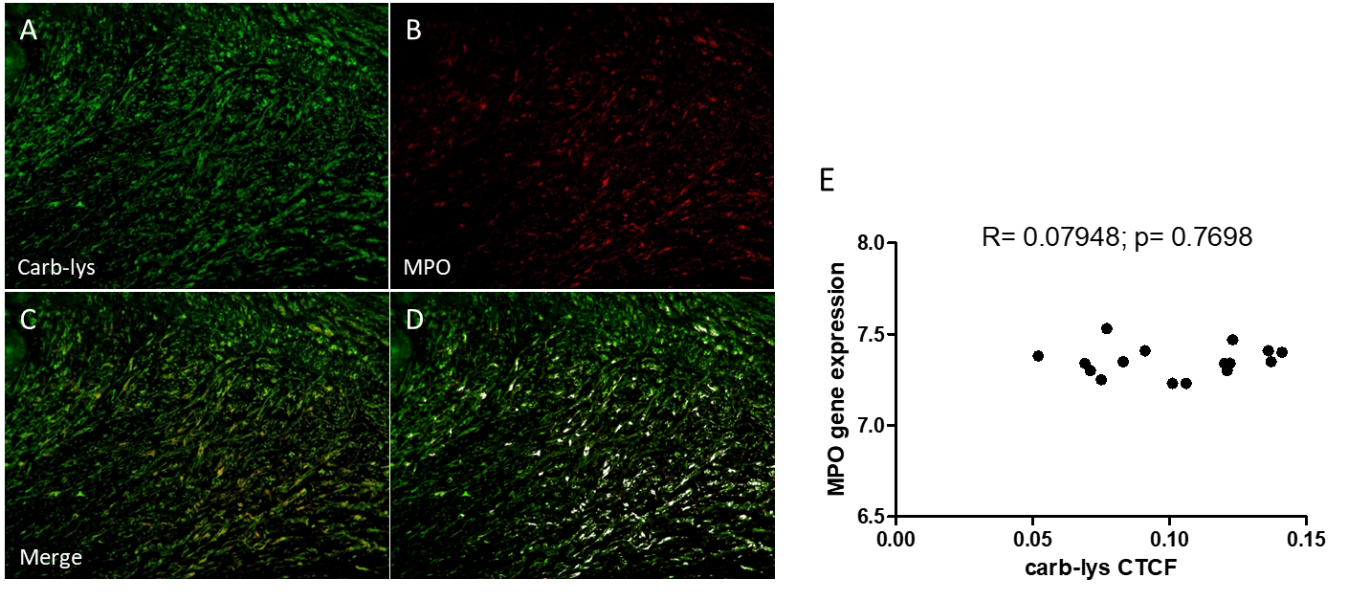
**

**Figure S5. MPO might not be the only source of carbamylation in the plaque.** **A**-**D**) Pseudo-fluorescent images showing carb-lys, MPO and merged channels as well as merged image with highlighted areas of co-localization (white areas, **D**). **E**) Correlation between carb-lys signal (median CTCF) and MPO gene expression in the plaque samples of the MaasHPS sub-cohort. MPO expression values extracted from the publicly available data (Jin *et al*., 2021).


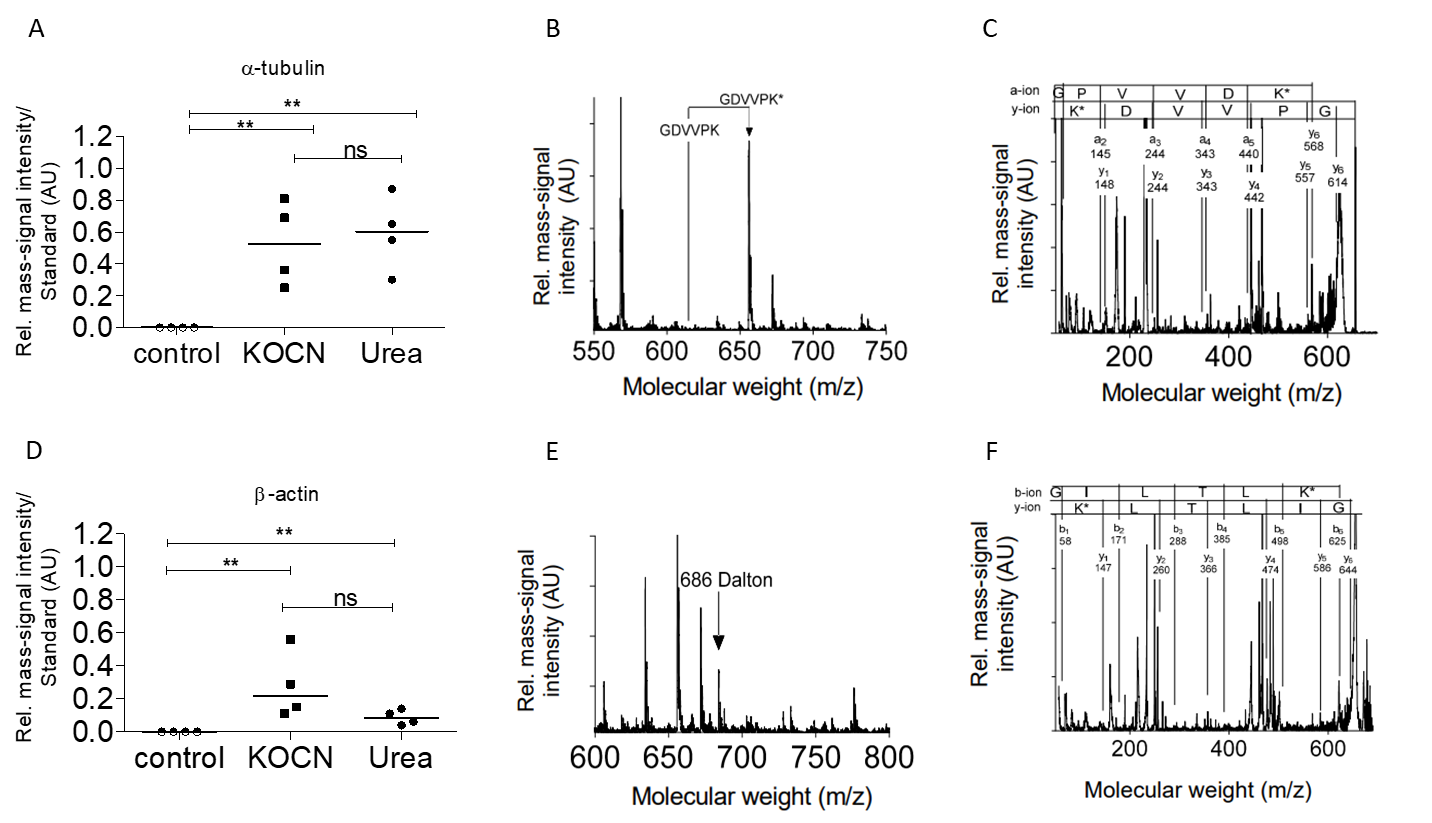


**Figure S6. Mass spectrometry detects increased carbamylation of α-tubulin and β-actin** **after macrophage treatment with KOCN or urea**. THP-1 derived macrophages were treated with 5 mM KOCN or 40 mM urea for 24 hours. Protein lysates were separated on SDS-PAGE and protein bands ~38-42 kD and 50-55 kD were analyzed by MALDI-TOF-TOF. **A**) and **D**) Quantification of carbamylated α-tubulin and β-actin peaks, respectively. **B**) and **E**) Representative MALDI-MS spectra of α-tubulin and β-actin, resp. **C**) and **F**) Representative MALDI-MS/MS spectra of α-tubulin and β-actin, resp.

**
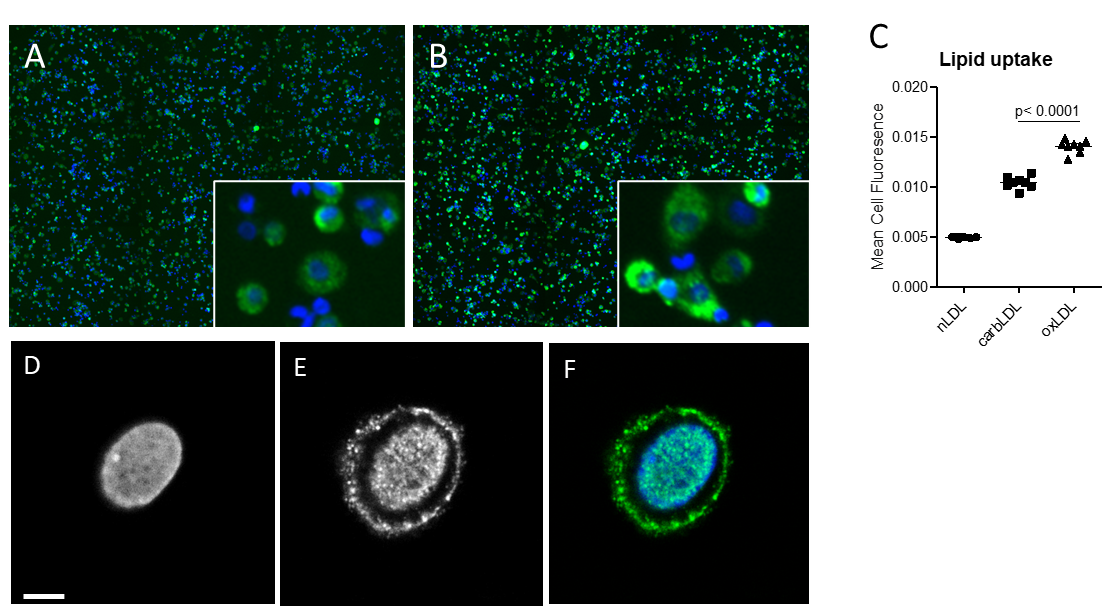
**

**Figure S7. Short-term uptake of carbLDL by macrophages is lower compared to that of oxLDL.** **A**-**B**) representative images of the lipid uptake analysis of THP-1 cells treated with carbLDL (**A**) and oxLDL (**B**). **C**) Quantification of the Topfluor signal. **D**-**F**) Pilot experiment using confocal imaging on human macrophages derived from peripheral blood mononuclear cells treated with 50 μg/ml carbLDL for 24 hours, showing DAPI (**H**), carb-lys (**I**) and merged (**J**) channels, n= 1. Size bars – 5 µm.
